# Comparison of alternative integration sites in the chromosome and the native plasmids of the cyanobacterium *Synechocystis* sp. PCC 6803 in respect to expression efficiency and copy number

**DOI:** 10.1186/s12934-021-01622-2

**Published:** 2021-07-10

**Authors:** Csaba Nagy, Kati Thiel, Edita Mulaku, Henna Mustila, Paula Tamagnini, Eva-Mari Aro, Catarina C. Pacheco, Pauli Kallio

**Affiliations:** 1 Molecular Plant Biology, Department of Life Technologies, University of Turku, Itäinen Pitkäkatu 4 C, 20520 Turku, Finland; 2 i3S-Instituto de Investigação e Inovação em Saúde, Universidade do Porto, Rua Alfredo Allen, 208, 4200-135 Porto, Portugal; 3 IBMC-Instituto de Biologia Molecular e Celular, Universidade do Porto, Rua Alfredo Allen, 208, 4200-135 Porto, Portugal; 4 Departamento de Biologia, Faculdade de Ciências, Universidade do Porto, Rua do Campo Alegre, Edifício FC4, 4169-007 Porto, Portugal

**Keywords:** *Synechocystis* sp. PCC 6803, Genomic integration, sYFP2, Native plasmids, Replicon copy number

## Abstract

**Background:**

*Synechocystis* sp. PCC 6803 provides a well-established reference point to cyanobacterial metabolic engineering as part of basic photosynthesis research, as well as in the development of next-generation biotechnological production systems. This study focused on expanding the current knowledge on genomic integration of expression constructs in *Synechocystis*, targeting a range of novel sites in the chromosome and in the native plasmids, together with established loci used in literature. The key objective was to obtain quantitative information on site-specific expression in reference to replicon copy numbers, which has been speculated but never compared side by side in this host.

**Results:**

An optimized sYFP2 expression cassette was successfully integrated in two novel sites in *Synechocystis* chromosome (*slr0944*; *sll0058*) and in all four endogenous megaplasmids (pSYSM/*slr5037*-*slr5038*; pSYSX/*slr6037*; pSYSA/*slr7023*; pSYSG/*slr8030*) that have not been previously evaluated for the purpose. Fluorescent analysis of the segregated strains revealed that the expression levels between the megaplasmids and chromosomal constructs were very similar, and reinforced the view that highest expression in *Synechocystis* can be obtained using RSF1010-derived replicative vectors or the native small plasmid pCA2.4 evaluated in comparison. Parallel replicon copy number analysis by RT-qPCR showed that the expression from the alternative loci is largely determined by the gene dosage in *Synechocystis*, thereby confirming the dependence formerly proposed based on literature.

**Conclusions:**

This study brings together nine different integrative loci in the genome of *Synechocystis* to demonstrate quantitative differences between target sites in the chromosome, the native plasmids, and a RSF1010-based replicative expression vector. To date, this is the most comprehensive comparison of alternative integrative sites in *Synechocystis*, and provides the first direct reference between expression efficiency and replicon gene dosage in the context. In the light of existing literature, the findings support the view that the small native plasmids can be notably more difficult to target than the chromosome or the megaplasmids, and that the RSF1010-derived vectors may be surprisingly well maintained under non-selective culture conditions in this cyanobacterial host. Altogether, the work broadens our views on genomic integration and the rational use of different integrative loci versus replicative plasmids, when aiming at expressing heterologous genes in *Synechocystis*.

**Supplementary Information:**

The online version contains supplementary material available at 10.1186/s12934-021-01622-2.

## Introduction

Microorganisms are routinely engineered by introducing recombinant genetic material into the cell to express individual target proteins, produce specific metabolites, or to study enzyme functions in vivo. This can be accomplished by either integrating the expression cassette into the host genome using, for example, homologous recombination, site-specific recombination or transposase-mediated gene transposition depending on the organism, or as part of a replicative plasmid [1, 2]. A typical objective is to generate a system which is genetically stable and allows regulated protein expression at a wide dynamic range, without unwanted adverse effects to the host. In integrative systems, the challenge is to find a redundant genomic locus (*neutral site*), that can be disrupted without compromising native functions that would affect maintenance and cell growth [3], or a gene of known function that is not required under the specific cultivation conditions used. Applicable target sites are therefore strain-specific, and linked with the experimental setup, that may influence production efficiency and system stability [4]. In addition, in polyploid organisms such as the model cyanobacterium *Synechocystis* sp. PCC 6803 (*Synechocystis* from here on) studied here, the introduced gene cassette must segregate in all parallel copies of the chromosome to prevent emergence of wild type progeny through negative selection. A critical factor affecting the level of expression is the relative copy number of the replicon, chromosome or plasmid, that determines the number of target genes in each cell (*gene dosage effect*) [5]. The copy numbers change from host to another, and may vary remarkably depending on the growth phase and environmental conditions, as observed in *Synechocystis* [6, 7] (see Tables 1, 2). In addition, genomic locus-specific effects that relate to DNA organization and replication with localized effects on transcriptional efficiency (*chromosomal position effect*) have a significant impact on the expression levels in some microorganisms such as *E. coli* and *S. cerevisiae* [5, 8, 9]. These factors essentially depend on the organism, and understanding them is important for the rational design of expression systems in basic and applied research.

**Table 1:** Chromosome copy numbers previously reported for different *Synechocystis* (sub)strains

*Synechocystis* (sub)strain	OD_750_ or growth phase	Chromosome copy numbers per cell	Method	Reference
GT^a^	0.1/0.6/Stat	142/47/43	RT-qPCR	[31]
GT-P^b^	0.3–0.5	9	Flow cytometry	[79]
	0.1/7.0	6/2.5	Flow cytometry	[80]
GT-W^c^	0.3–0.5	10	Flow cytometry	[79]
	0.1/0.5/2.5	22/18/6	RT-qPCR	[6]
	0.5	21	Spectroscopic	
(GT-)Kazusa	1.0 (A_580_)	12	Colorimetric	[81]
	0.1/0.5/2.5	14/7/1	RT-qPCR	[6]
	0.5	10	Spectroscopic	
(GT-)Michel^d^	0.1/0.5/2.5	19/15/8	RT-qPCR	[6]
	0.5	22	Spectroscopic	
PCC-M^e^	0.1/0.6/Stat	218/58/58	RT-qPCR	[31]
	0.1/0.5/2.5	23/18/4	RT-qPCR	[6]
	0.5	18	Spectroscopic	
	N.A.	5	Flow cytometry	[82]
(PCC-)Rippka	0.1/0.5/2.5	21/6/6	RT-qPCR	[6]
	0.5	15	Spectroscopic	
(PCC-)Hagemann	0.1/0.5/2.5	19/12/4	RT-qPCR	[6]
	0.5	20	Spectroscopic	
N.A.	Exp	5–6	Flow cytometry	[83]
N.A.	~ 5.0	11	RT-qPCR	[42]

^a^ GT: glucose tolerant strain

^b^ GT-P: GT strain obtained from the laboratory of Prof. Peter Nixon (Imperial College London)

^c^ GT-W: GT strain obtained from the laboratory of Prof. Wim Vermaas (Arizona State University)

^d^ (GT-)Michel: GT strain obtained from the laboratory of Prof. Klaus-Peter Michel (Bielefeld University)

^e^ PCC-M: Pasteur Culture Collection motile strain obtained from the lab of Prof. Sergey Shestakov (Moscow State University)

N.A.: Not assigned, Stat: Stationary phase; Exp: Exponential phase

**Table 2:** Relative and/or absolute copy numbers of replicative plasmids in *Synechocystis* reported in the literature

	Origin of replication	OD_750_	Copy number/chromosome	Copy number/cell	Reference
Native plasmids					
pSYSA	N.A.	0.25/2.25	0.34/0.33	N.A.	[84]
		1.5	1.61	N.A.	This study
pSYSG	N.A.	0.25 /2.25	0.64/0.54	N.A.	[84]
		1.5	1.15	N.A.	This study
pSYSM	N.A.	0.25/2.25	0.33/0.31	N.A.	[84]
		1.5	1.72	N.A.	This study
pSYSX	N.A.	0.25/2.25	0.65/0.66	N.A.	[84]
		1.5	1.85	N.A.	This study
pCC5.2	N.A.	0.25/2.25	0.93/3.72	N.A.	[84]
pCA2.4	N.A.	0.25/2.25	0.75/5.41	N.A.	[84]
		1.5	5.36	N.A.	This study
pCB2.4	N.A.	0.25/2.25	0.40/2.46	N.A.	[84]
Non-native plasmids					
pSB2A	RSF1010	N.A.	N.A.	10	[85]
pFC1	RSF1010	N.A.	N.A.	10	[86]
pMB13	RSF1010	N.A.	N.A.	10	[86]
pSL1112	RSF1010	N.A.	3.3	29	[87]
pPMQAK1	RSF1010	5	N.A.	31	[42]
		2.6	1	N.A.	[44]
pDF-lac2	RSF1010	1.5	2.39	N.A.	This study
pSEVA421	RK2	5	N.A.	9	[42]
pSCB	From pCC5.2	2.6	10	350–500	[44]

*N.A.* Not assigned

Cyanobacteria are a diverse group of ancient photoautotrophic prokaryotes and the progenitors of oxygenic photosynthesis on Earth [10]. They contribute significantly to the global primary production, and have an indispensable role in maintaining functional aquatic ecosystems and biogeochemical cycles by regulating global carbon and nitrogen turnover [12]. Due to the relative simplicity of the bacterial system in comparison to plants and green algae, cyanobacteria have been used as model organisms to solve the basic principles of oxygenic photosynthesis, including the structure, function and organization of the large protein complexes operating in water oxidation and electron transfer reactions [12–15] as well as the mechanisms for maintenance of carbon/nitrogen homeostasis [16]. In addition, the regulatory systems related to photoprotection and the maintenance of the photosynthetic machinery under changing environmental conditions, which differ substantially from both plants and algae, have been extensively investigated in cyanobacteria [17–19]. Due to the inherent ability to utilize solar energy for the generation of diverse organic molecules from CO_2_, cyanobacteria have also been recognized as potential next-generation biotechnological hosts for the production of commercially interesting compounds such as platform chemicals, polymer precursors and biofuels [20–22]. The most extensively studied cyanobacterium is the unicellular freshwater species *Synechocystis*, for which many distinct wild type substrains have been actively used [23–25]. Despite the increasing interest in expanding towards alternative strains with specific physiological properties such as faster growth rate or tolerance to high light intensities and salinity [26, 27], *Synechocystis* continues to have an important role in many leading laboratories due to the wealth of existing biological information, synthetic biology tools and technical know-how [28–30]. From the perspective of the current work, considerations that relate to the rational engineering of *Synechocystis* genome include: (i) polyploidy of the chromosome (Table 1) which complicates the preparative steps due to time-consuming strain segregation, (ii) differences between putative integration sites that may affect system stability due to site-specific functions, (iii) expression efficiency that is related to the copy number of the target replicon, and (iv) the choice between available replicative plasmids offering alternatives to genomic integration.

The *Synechocystis* genome consists of a single polyploid chromosome (3.57 Mb) [6, 31], four different megaplasmids (pSYSM/120 kb; pSYSX/106 kb; pSYSA/103 kb; pSYSG/44 kb) [32], and three small plasmids (pCC5.2/5.2 kb; pCA2.4/2.4 kb; pCB2.4/2.4 kb) [33–35]. So far almost all the genomic integration sites used for expressing heterologous genes in *Synechocystis* reside specifically in the chromosome, including slr1311* (*psbA2*) and slr0168* (N.A.) that are amongst the most common neutral sites referred to in literature (Table 3). In comparison, only one of the native plasmids, pCA2.4, has been successfully recruited for heterologous expression (36) (Table 3). Besides integration, autonomous plasmids based on the broad-host-range replicon RSF1010 [37] such as pFC1 [38], pPMQAK1 [39], pDF [40], and pSEVA*n*5*n* [41], or the pSEVA421 plasmid derived from RK2 [42] have also been applied in *Synechocystis*. In addition, recent expression vectors generated based on the endogenous plasmids pCA2.4, pCB2.4 and pCC5.2 [43–45] provide alternatives for engineering. Despite the collective information on the use of integrative constructs and replicative plasmids in *Synechocystis* (Table 3), direct quantitative data on the relative expression efficiencies between the chromosome, the native megaplasmids and RSF1010-based vectors does not exist. In addition, the corresponding copy number data has never been collected in parallel, and there is rather distinct variation between different experimental setups described in literature which is known to affect the acquired copy numbers (Tables 1, 2), thereby complicating direct comparison. We therefore anticipated that further characterization could provide useful insight for future engineering, when, for example, maximizing expression or evaluating the pros and cons between different alternatives for integration.

**Table 3:** Genomic neutral sites used earlier for the integration of expression cassettes in *Synechocystis*

Integration site	Replicon	Expressed protein/source organism	Reference
slr1311* (*psbA2*)	Chrom	Ipi/*S. cerevisiae*; CrtR, CrtP, CrtB/*Synechocystis* Pdc, Adh/*Z. mobilis* IspS/*P. montana* IspS/*P. montana* HmgS, HmgR/*E. faecalis*; AtoB/*E. coli* YFP	[88] [89] [59] [90] [91] [36]
slr0168* (N.A.)	Chrom	GFP/*A. victoria*, LuxAB/*V. harveyi* Ldh/*B. subtilis*, Sth/*P. aeruginosa* GFPmut3B	[92] [60] [93]
*slr1181* (*psbA1*)	Chrom	Kivd, AdhA/*L. lactis*	[94]
*sll1476* (N.A.)	Chrom	GFP/*A. victoria*	[65]
*slr0573* (N.A.)	Chrom	GFP/*A. victoria*	[65]
*slr1396* (N.A.)	Chrom	GFP/*A. victoria*	[65]
*slr0271* (N.A.)	Chrom	GFP/*A. victoria*	[65]
*slr0397* (N.A.)	Chrom	GFP/*A. victoria*	[65]
I.R.*	pCA2.4	YFP	[36]

N.A.: Not assigned; gene encoding protein of unknown function or annotated as hypothetical; Chrom.: Chromosome; I.R.*: Intergenic region between *slr9101* and *pCA24_1* (position 227—465 bp) in pCA2.4.; Ipi: isopentenyl diphosphate isomerase; CrtR: β-carotene hydroxylase; CrtP: phytoene desaturase; CrtB*:* phytoene synthase; Pdc: pyruvate decarboxylase; Adh: alcohol dehydrogenase II; IspS: isoprene synthase; HmgS: Hmg-CoA synthase; HmgR: Hmg-CoA reductase; AtoB: acetyl-CoA acetyl transferase; GFP*:* green fluorescent protein; LuxAB: luciferase; Ldh*:* L-lactate dehydrogenase; Sth: soluble transhydrogenase; GFPmut3B: synthetic green fluorescent protein; Kivd: 2-ketoisovalerate decarboxylase; AdhA: aldehyde reductase; YFP: yellow fluorescent protein. The integration sites used as controls in this study have been indicated by *

From these premises, we set off to compare a set of novel genomic target sites against established loci for expressing recombinant proteins in *Synechocystis*, with specific emphasis on the native megaplasmids that have not been utilized for this purpose earlier. The hypothesis was that the comparison would expand our practical knowledge on the alternative integrative sites in regards to efficiency, but possibly also in copy number, construct stability or chromosomal position effects. The approach was to integrate and monitor the expression of yellow fluorescent protein (sYFP2) in different selected loci in *Synechocystis* chromosome and the native plasmids, thereby allowing the quantitative comparison of expression and relative gene dosage in reference to the pDF and pCA2.4 plasmid-based systems.

## Results

### Selecting candidate integration sites in the genome of *Synechocystis*

**Fig. 1:**
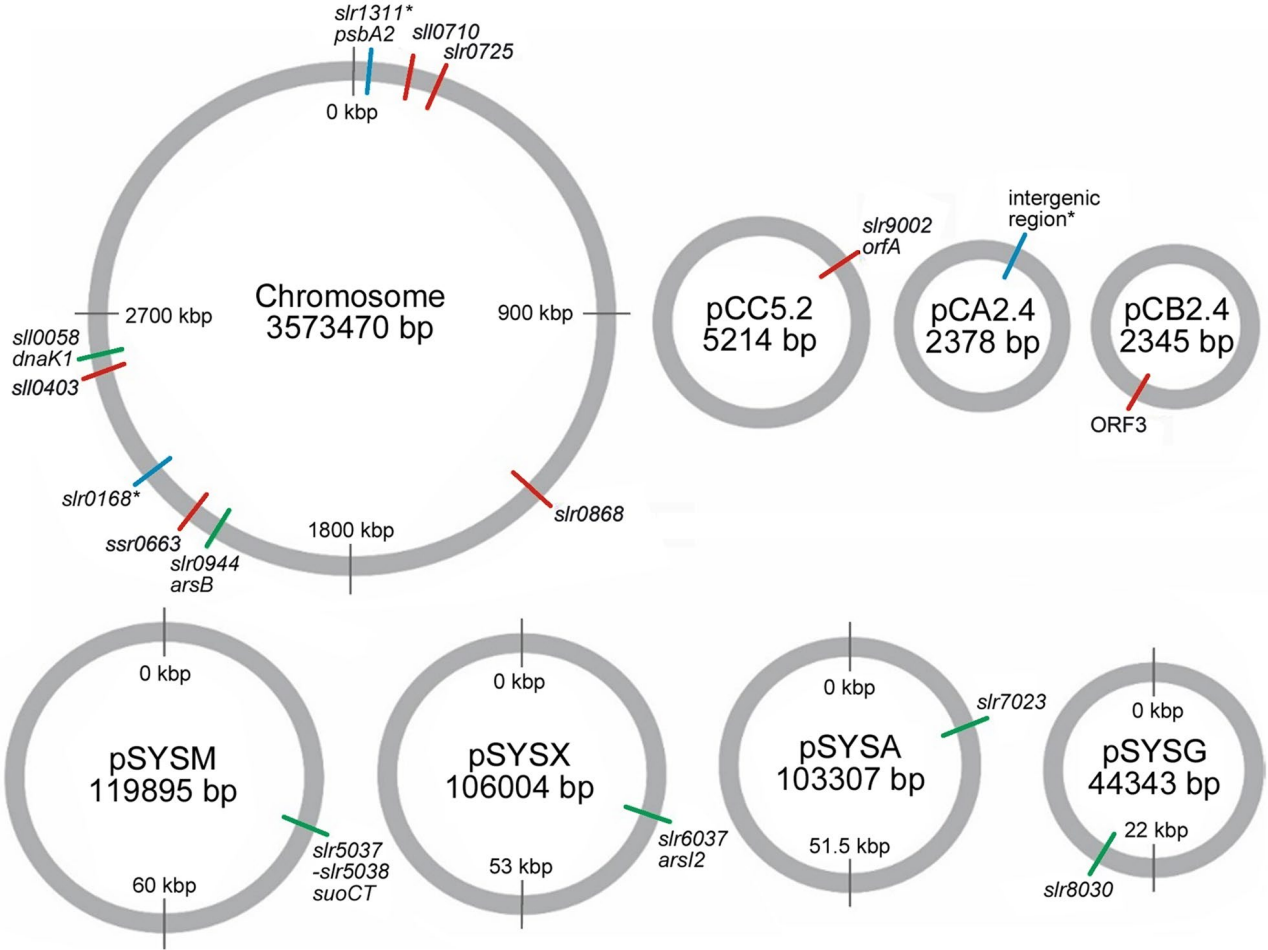
Schematic representation of the genome of *Synechocystis* showing the integration sites in the chromosome and in the native plasmids targeted in this study. The figure depicts the novel functional integration sites characterized in this work (green lines), the loci previously used for engineering purposes that serve as quantitative controls (blue lines; *), and the sites which failed to produce stable integration mutants (red lines). See Table 4. for details

To compare alternative integration sites in *Synechocystis* in terms of expression level and stability, 16 genomic loci (Fig. 1; Table 4) were selected as candidates for the integration of an optimized sYFP2 expression cassette [46, 47] (Additional file 1: Figs. S1, S2). Out of these, 13 target sites had not been previously used for engineering, and included seven novel loci in the chromosome (*sll0710*, *slr0725*, *slr0868*, *slr0944*, *ssr0663*, *sll0403*, *sll0058*), one in each of the four megaplasmids (pSYSM/*slr5037*-*slr5038*; pSYSX/*slr6037*; pSYSA/*slr7023*; pSYSG/*slr8030*), and two in the small native plasmids (pCC5.2/*slr9002*; pCB2.4/*ssr9202*). Five of the new chromosomal target sites were genes of unknown function (*sll0710*, *slr0725*, *slr0868*, *ssr0663*, *sll0403*), chosen primarily based on minimal condition-specific expression level changes in genome-wide transcriptomic analysis (Additional file 1: Table S1; see "Materials and methods" for details). The screening relied on pre-existing high-quality microarray data, which was originally acquired for *Synechocystis* WT and photosynthetic electron transfer deletion strains upon the transition from ambient CO_2_ levels to high carbon (3%), and from constant light to fluctuating light [48, 49]. These conditions were considered relevant from biotechnological viewpoint, as they represent the use of elevated CO_2_ concentrations to enhance productivity, and the effects of light fluctuation in large-scale cultures, as induced by shelf-shading and constantly varying distance from the light source. In addition, the deletion strains in the microarray study [48, 49] were of interest from engineering perspective, as the inactivation of native alternative electron sinks such as the flavodiiron proteins may provide means for enhancing the cellular metabolic flux towards specified end-products [50–52]. The two remaining chromosomal targets were *arsB* (*slr0944*) associated with arsenic resistance [53] and heat-shock gene homolog *dnaK1* (*sll0058*) [54] (Table 4), which based on the characterized functions were expected to be dispensable under standard culture conditions used for expression. In agreement, these two targets showed negligible transcript level changes also in the earlier microarray analysis under all the studied conditions and in all the strains (Additional file 1: Table S2). The candidate sites on the native megaplasmids, *suoCT* (*slr5037- slr5038*) in pSYSM [55] and *arsI2* (*slr6037*) in pSYSX [56] were also linked with the metabolism of arsenic compounds, whereas *slr7023* in pSYSA [57] and *slr8030* in pSYSG had no critical or essential functions identified so far (Table 4). Due to the compact nature of the small native plasmids, the options for target sites were rather limited, and the two uncharacterized genes *slr9002* (*orfA*) in pCC5.2 and *ssr9202* (ORF3) in pCB2.4 [35] were selected because they resided outside the regions that were previously shown to be indispensable [c.f. [58]]. Finally, to allow comparison with established integration sites used earlier, two chromosomal loci slr1311* [59] and slr0168* [60], and the intergenic neutral region between the genes *slr9101* and *pCA24_1* (ORF2) in pCA2.4/I.R.* [36] were included as reference controls (indicated by asterisks throughout).

**Table 4:**
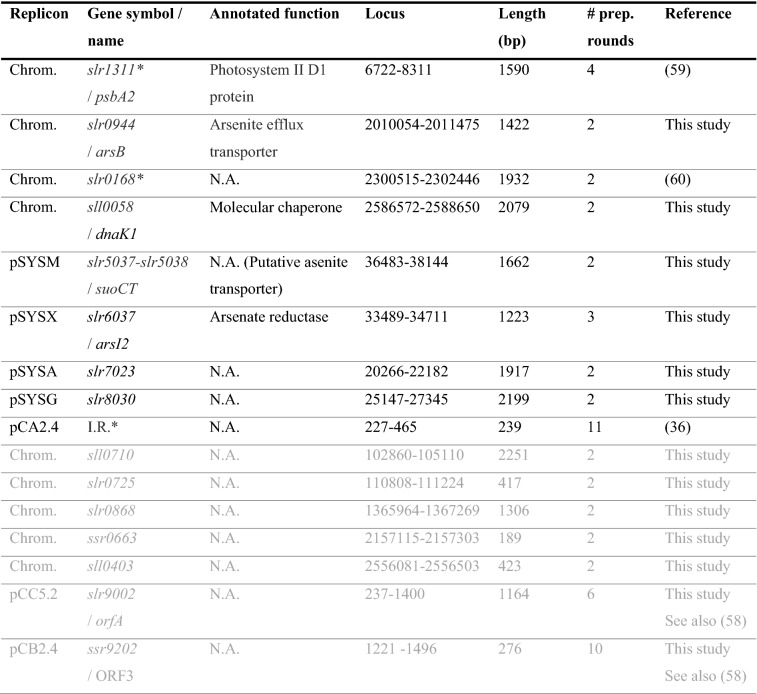
List of the genomic integration sites in *Synechocystis* targeted in this study. The integrative sites which were successfully used for the introduction of an sYFP2 expression cassette are shown in black font. The strains that were unstable or could not be segregated (and were excluded from the subsequent analysis) are shown in grey font. The sites previously reported in literature that were used as quantitative controls have been indicated by * [the control strain harboring the replicative pDF-lac2* plasmid is not listed in the table]

*N.A.* Not assigned, gene encoding protein of unknown function or annotated as hypothetical, *Chrom.* Chromosome; I.R.* Intergenic region between *slr9101* and *pCA24_1* (position 227–465 bp) in pCA2.4. # prep. rounds. The number of independent transformation /segregation attempts conducted for each strain

### Generation of nine *Synechocystis* strains expressing sYFP2 from different genomic loci

The sYFP2 expression cassette (Additional file 1: Fig. S2) was introduced into each selected site in *Synechocystis* genome (Fig. 1, Table 4) using an integrative vector pSI1B (Additional file 1: Fig. S1) generated in this work. The pSI1B vector is based on the commercial plasmid pUC57, and was designed to integrate into the genome by homologous recombination using PCR-amplified flanking sequences specific to each of the target loci (see Additional file 1: Table S3). The constructed plasmids (Additional file 1: Table S4) were transformed into *Synechocystis*, followed by selection on increasing concentrations of antibiotics, and analysis by colony PCR to verify the resulting recombinant strains (Additional file 1: Fig. S3). As preparative efficiency was one of our selection criteria, the candidate strains were first subjected to two rounds of transformation and segregation. In the case of the novel chromosomal target sites, the resulting positive strains qualified to the subsequent analytical phase of the work, while those that were unsuccessful were excluded (see Table 4). Additional preparative rounds were performed to obtain the remaining reference strains, as well as the missing strains expressing sYFP2 from the newly-selected target loci in the native plasmids, as they were of particular interest in this study. As the outcome, nine different *Synechocystis* strains were constructed, harboring the sYFP2 expression casette in two novel chromosomal loci (*slr0944* and *sll0058*)*,* all four native megaplasmids (pSYSM/*slr5037*-*slr5038*; pSYSX/*slr6037*; pSYSA/*slr7023*; pSYSG/*slr8030*), and three control loci (slr1311*, slr0168*, pCA2.4/I.R.*) (Table 4; black font, Additional file 1: Fig. S3). Notably, as seen from Table 4, many of the chromosomal and the megaplasmid target sites could be disrupted with relative ease, while the small plasmids pCB2.4/*ssr9202* and pCC5.2/*slr9002* did not yield positive hits even after six and ten independent preparative rounds, respectively. In the case of pCB2.4/*ssr9202,* colony PCR bands corresponding to the correct integrative constructs were never observed, whereas for pCC5.2/*slr9002* the detected bands were either the wrong size, or the strains could not be further segregated. The additional reference strain carrying the replicative plasmid pDF-lac2* with the same sYFP2 expression cassette was generated and verified earlier [47].

### Fluorescence profiles reveal expression-level differences between the engineered *Synechocystis* strains

**Fig. 2:**
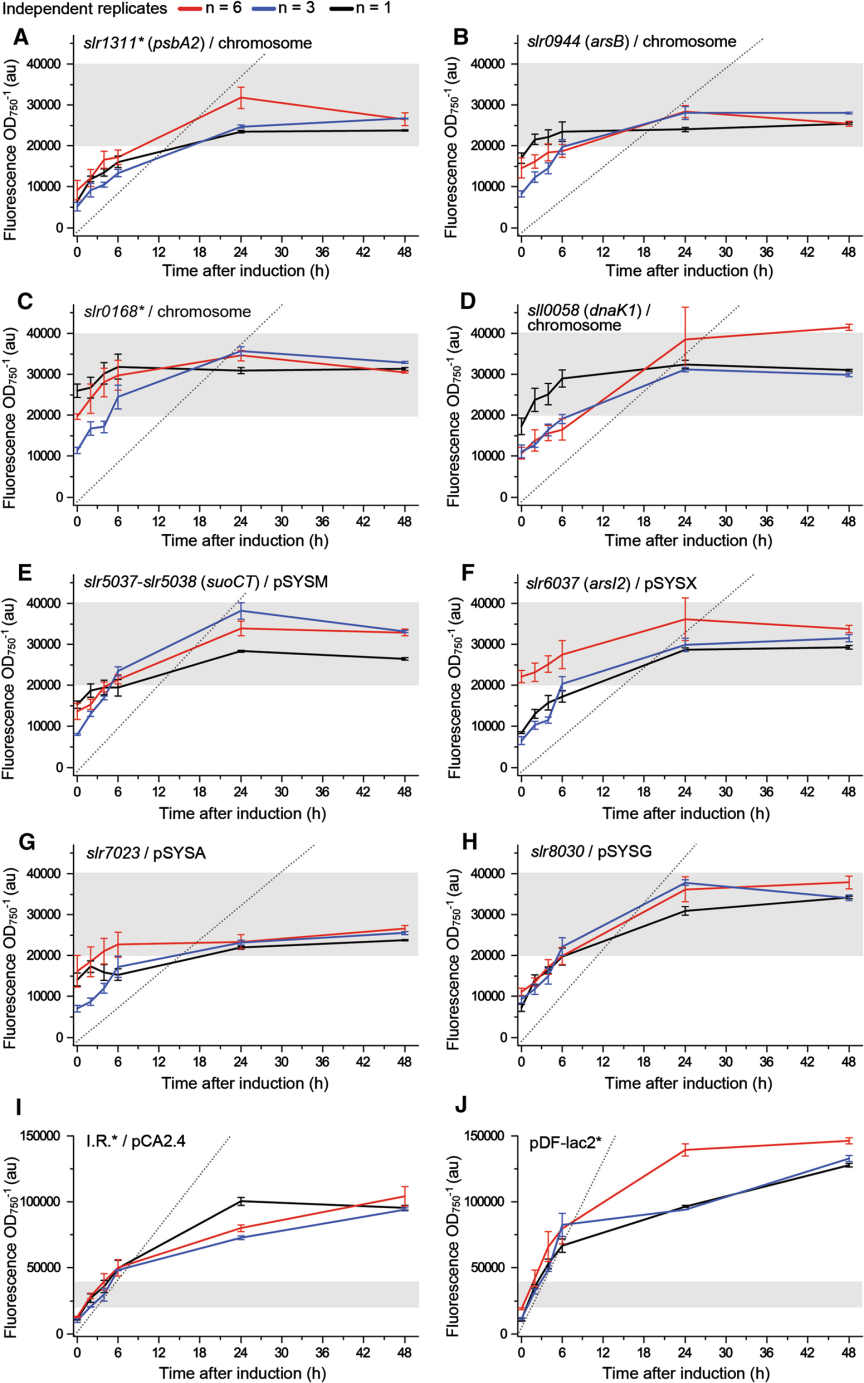
Quantitative analysis of the generated *Synechocystis* expression strains, showing the sYFP2 fluorescence signal recorded at the timepoints 0, 2, 4, 6, 24 and 48 h after induction with 1 mM IPTG The experiment was repeated three times, using six independent replicates (red line; n = 6), three independent replicates ( blue line; n = 3), or a single culture (black line; n = 1) for each strain **A**–**J**. The values were always measured in three technical replicates, normalized to OD_750_, and used for calculating the plotted averages and the standard deviations. The initial rates of fluorescence increase, calculated based on three most consistent data points between 0–6 h and averaged between the three parallel runs, have been shown in grey dotted lines. The horizontal grey bar shows the 20,000–40,000 fluorescence level range to visualize the scale difference between **A**–**H** and **I**, **J**. The control strains have been indicated by *. Negative WT reference is presented in Additional file 1: Fig. S4.

To compare the generated *Synechocystis* strains (Table 4) in regards to expression efficiency, the cells were cultured and analyzed for sYFP2 fluorescence at six successive timepoints (0, 2, 4, 6, 24 and 48 h) after induction. The resulting fluorescent profiles (Fig. 2, Additional file 1: Fig. S4) displayed strain-specific expression dynamics throughout the induction phase, thereby providing a basis for more reliable comparison than would be possible with a single end-point assay. The analysis was divided in three independent experiments to evaluate system reproducibility, and to avoid practical constraints in processing large numbers of cultures at specified sampling points. In the first series (Fig. 2; red line), the ten strains were characterized one at a time using six independent replicates (n = 6). This was done to determine the level of biological variance between parallel samples, and to establish reference expression profiles for subsequent comparisons. In the following two series (Fig. 2; blue and black lines) the ten strains were characterized all at the same time but with lower number of replicates (n = 3 and n = 1). This allowed us to critically assess between the actual strain-specific differences, and the technical variation from one experiment to another.

**Fig. 3:**
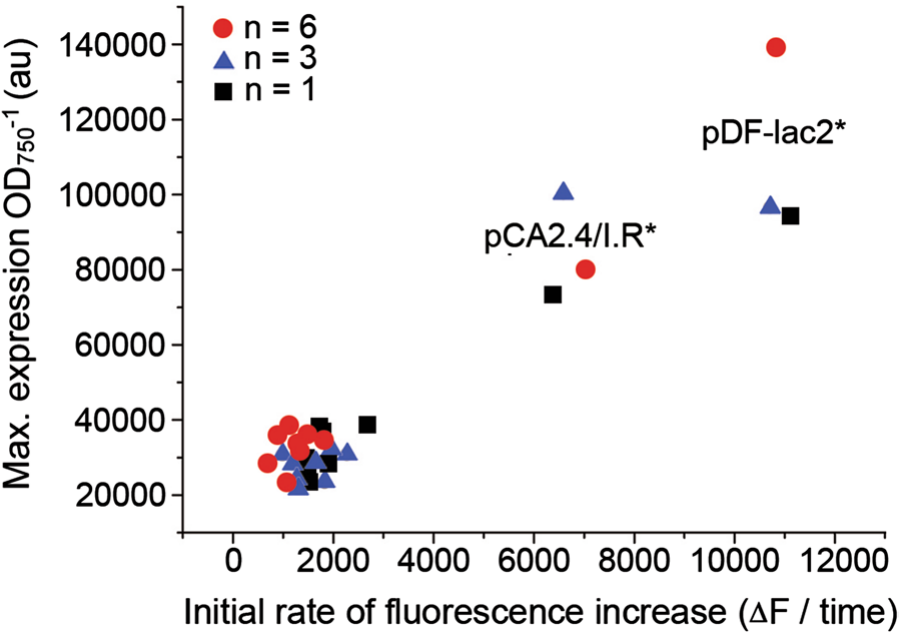
Maximum sYFP2 expression levels versus the initial expression rates recorded for the different integrative constructs in *Synechocystis* (see Table 4. for strain details). The data represents three independently conducted experiments using different number of biological replicates as shown in red circle (n = 6), blue triangle (n = 3) and black square (n = 1). The relative maximum fluorescence values were recorded at the 24 h timepoint after induction, and the expression rates calculated from the linear fits between the first three or four measurement points after induction presented in Fig. 2.

**Fig. 4:**
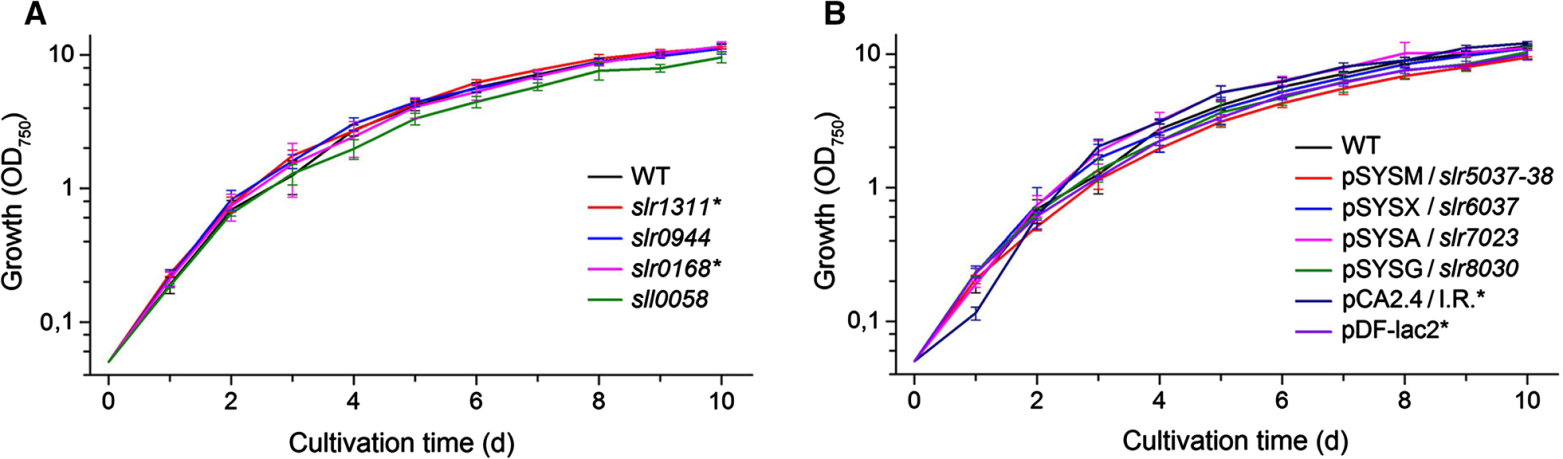
Growth of the generated *Synechocystis* strains harboring the sYFP2 expression cassette in the A) chromosome or in the B) native plasmids, in reference to the WT and the control strains (*). The cultivations were carried out in Erlenmeyer flasks, at 30 °C under constant light of 50 μmol photons m^−2^ s^−1^ and 1% CO_2_ atmosphere, and the growth was measured as OD_750_ increase over a period of seven days from induction. The mean and standard deviation at each measurement point have been calculated from three independent cultivations

Despite small differences between the last two measuring points in some cases, the fluorescent profiles (Fig. 2) suggested that the 24 h timepoint would be a reliable estimate for the maximum fluorescence levels (*i.e.* maximum expression efficiency), and a basis for evaluating the expression capacity between the parallel loci. The maximum expression efficiency between the chromosomal sites (Fig. 2A–D) and the megaplasmids (Fig. 2E–H) were all within a similar range (in between 20,000–40,000 fluorescence a.u). In contrast, the recorded values were significantly higher for the small native plasmid pCA2.4/I.R.* (Fig. 2I; 75,000–100,000 fluorescence a.u) and pDF-lac2* (Fig. 2J; 100,000–150,000 fluorescence a.u), corresponding approximately to two- and three-fold higher expression levels at the 24 h maximum, respectively. In support of these findings, comparison of the averaged initial expression rates, calculated based on the fluorescence increase over the first hours of induction (Fig. 2; grey dotted lines; see "Materials and methods" for details), resulted in a corresponding relative correlation between the strains as the data collected at the 24 h timepoint. This outcome was further visualized by plotting the initial rates against the maximum expression levels (Fig. 3), showing that most strains group together, with the exception of the pCA2.4/I.R.* and pDF-lac2* strains that appear as independent clusters. The growth of the engineered strains appeared consistent in comparison to the WT control (Fig. 4), and there was no apparent dependence between the OD_750_ profiles and the maximum expression levels, suggesting that the expression of sYFP2 did not cause metabolic stress that would compromise fitness of any of the strains under the experimental conditions used.

### Real-time quantitative PCR reveals differences in the s*yfp*2 copy number between chromosomal and plasmid-based replicons

To evaluate the relationship between the observed expression levels and gene dosage, the s*yfp*2 copy number in each generated strain was determined in reference to the number of the chromosome copies (*i.e.* normalized against the genes *petB* and *rrn16S*) by real-time quantitative PCR (RT-qPCR). Cell lysates were used as templates for the amplification reactions to minimize sample processing and possible variation in the extraction of chromosomal DNA and the plasmids. The integrity of the lysates was evaluated on agarose gel (Additional file 1: Fig. S5), which showed the presence of high-molecular weight DNA, fluorescent proteins (phycobiliproteins) and RNA in all samples. In addition, spectrophotometric analysis indicated similar sample composition based on the measured A_260_/A_280_ and A_260_/A_230_ ratios (SD_260/280_ = 0.07 and SD_260/230_ = 0.06, for more details see Additional file 1: Table S5). The primer pairs targeting the three analyzed genes showed acceptable amplification efficiencies (E) of ≈ 90% (Additional file 1: Table S6), which were in the range expected for complex protein-containing samples. The R^2^ values above 0.99 indicated that the optimized assay had a broad dynamic range, covering a series of five tenfold dilutions. In addition, the melt curve analysis confirmed amplicon specificity in all experiments (Additional file 1: Table S6; amplicon Tm and NTC), displaying a single sharp peak for each gene. Finally, to verify reaction specificity, the amplicon identity in each case was confirmed by DNA sequencing.

**Fig. 5:**
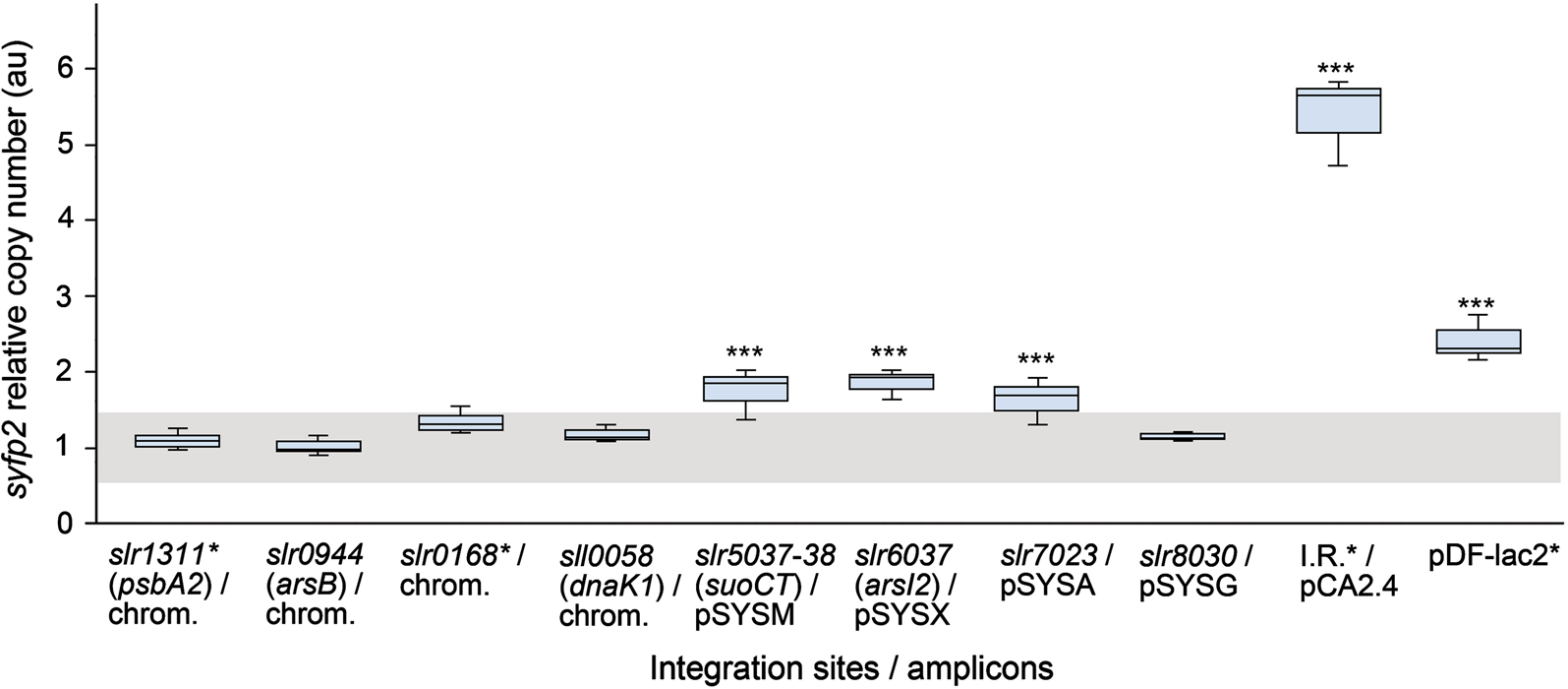
Determination of relative s*yfp*2 copy number for the different constructs introduced in *Synechocystis*. The s*yfp*2 copy numbers were determined for each strain in reference to the chromosome copy numbers (*i.e.* normalized against the endogenous genes *petB*, and *rrn16S*) by RT-qPCR. The *slr0944* (*arsB*) locus was selected as the control condition representing relative expression equal to one. The box-whisker plot represents three independent experimental trials with technical triplicates for all the strains (control strains indicated with *). One-way ANOVA test was performed to evaluate significant differences (*P* < 0.05 denoted by ***)

The RT-qPCR analysis indicated that the relative chromosome copy number did not significantly vary between the generated strains in reference to *petB* and *rrn16S*. This allowed these two genes to be used for s*yfp*2 normalization for determining the relative number of each of the target replicons. As anticipated, the relative copy numbers recorded for *syfp*2 in the different chromosome locations [slr1311* (*psbA2*), *slr0944* (*arsB*), *sll0058* (*dnaK1*) and slr0168*] were around one (measured values 1–1.3) (Fig. 5, Additional file 1: Table S5). The corresponding values for the megaplasmids pSYSM (*slr5037*-*slr5038*), pSYSX (*slr6037*), and pSYSA (*slr7023*) were slightly higher, ranging from 1.6 to 1.9, while for pSYSG it was 1.2. In comparison, the highest copy numbers were measured for the small plasmid pCA2.4 and the replicative vector pDF, corresponding to 5.4 and 2.4 per chromosome, respectively (Fig. 5, Additional file 1: Table S5).

### The engineered sYFP2 expression strains appear stable in a six-week cultivation

**Fig. 6:**
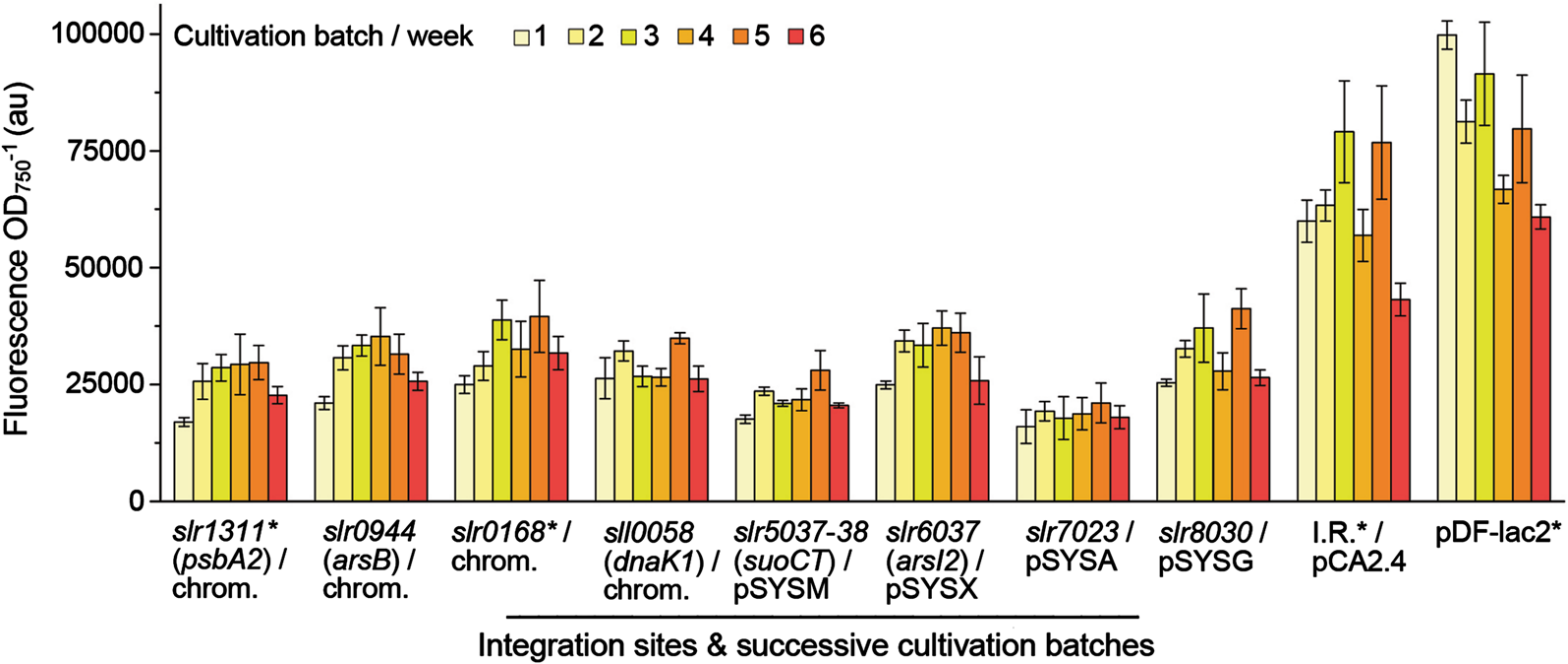
Long-term stability of the generated *Synechocystis* sYFP2 expression strains in the absence of antibiotic selection pressure. All the novel integration site mutants and the control strains (*) were monitored for sYFP expression in a step-wise suspension batch culture over a six-week period. The different colored bars represent the expression levels (normalized to OD_750_) measured at one-week intervals, 6 h after re-inoculation into a new batch with fresh BG-11 containing 1 mM IPTG and without antibiotics. The mean and standard deviation have been calculated from three independent replicate cultures (n = 3) for each strain at each timepoint

To evaluate the overall stability of the sYFP2-expressing strains, a series of successive batch cultivations was conducted without antibiotic selection pressure for a period of six weeks (Fig. 6). The cultures were re-inoculated at one-week intervals in fresh medium containing IPTG, followed by measurement of fluorescence six hours after induction. At each round, the axenity of the cultures was confirmed on antibiotic-free LB agar plates, and at the end of the trial, segregation was verified by construct-specific colony PCR (Additional file 1: Fig. S6). Despite some experimental fluctuation, the resulting fluorescence profiles showed that all the strains remained stable and maintained the capacity to express sYFP2 throughout the culture period, while the PCR screen confirmed that there were no obvious changes in the target sites at the DNA level. Notably, also the replicative pDF-lac2* construct appeared stable over the cultivation period of 42 days without antibiotic selection. To our knowledge, this is the longest reported time for monitoring expression plasmid maintenance in *Synechocystis*, and suggests that RSF1010-derived plasmids over-expressing essentially harmless proteins such as sYFP2 are not necessarily lost from the population as readily as could be anticipated.

## Discussion

Various genomic integration sites have been used for the expression of heterologous genes in *Synechocystis* to study native cellular functions, and to find the best alternatives for the production of specific chemical compounds (Table 3). To expand the existing knowledge, we targeted sites in *Synechocystis* chromosome, the native small plasmids and the four megaplasmids, and compared them in reference to known integration sites in the chromosome, the small plasmid pCA2.4 (Fig. 1), and RSF1010-based replicative plasmid pDF. Out of 13 initial candidate integration sites that were selected based on literature and existing microarray data, six novel loci were successfully targeted by sYFP2 to generate corresponding segregated mutant strains (Table 4; black font, Additional file 1: Fig. S3). As strain construction is typically time-consuming and a key limiting step in *Synechocystis* metabolic engineering, one of the initial evaluation criteria was the preparative efficiency, *i.e.* the relative ease at which the mutants could be obtained. Three out of four (3/4) megaplasmid target sites and two out of seven (2/7) novel chromosomal sites were successfully disrupted over the first two rounds of transformation and segregation, suggesting that the criteria applied for identifying alternative integration loci met the purpose. In contrast to these, targeting the selected sites in the three small native plasmids pCA2.4*, pCC5.2 and pCB2.4 was significantly more challenging (Table 4). The pCA2.4/I.R.* [36] control was successful only after 11 independent attemps (see "Materials and methods" for details), whereas the other two targets pCC5.2/*slr9002* and pCB2.4/*ssr9202* were excluded after six and ten futile preparative transformation-segregation rounds, respectively. Most earlier attempts to target the small plasmids at different loci in *Synechocystis* have also been unsuccessful [58, 36], which has been proposed to primarily result from the higher relative copy numbers of these replicons. So far pCA2.4/I.R.* has been the only plasmid-based integration site that has been disrupted and segregated [36], but with clearly lower preparative efficiency in comparison to the chromosome (slr1311*), as observed here. For our targets, repeated attempts to obtain correct transformants implicated that besides the segregation, there were also potential problems in homologous recombination and clone selection, yet the underlying molecular determinants remain unclear. These findings support the conclusions that the potential benefits in employing the small native plasmids for high-level expression in *Synechocystis* may be outweighed by critically low preparative efficiency, as compared to the chromosome or the megaplasmids. As an alternative, the use of replicative plasmids such as pDF-lac2* does not require segregation, so obtaining the corresponding expression strains is typically more straightforward.

The qualifying segregated strains did not show any obvious growth defects in reference to the WT (Fig. 4), suggesting that the deletions at the target loci did not inflict any unwanted metabolic responses that would compromise host fitness, and interfere with the following comparison. The functional analysis was based on the fluorescent marker sYFP2 [61], which enables sensitive non-invasive quantitation of gene expression, without inducing global toxicity effects. While well-suited for comparing expression efficiencies in vivo, fluorescent markers offer very limited information on expression-related adverse effects, and the resulting host response towards negative selection. More severe metabolic stress is induced by expression systems that deplete the cell from essential metabolic precursors or enzyme cofactors [60, 62], accumulate reactive pathway intermediates [63] or produce toxic end-products [64]. Such effects may be lethal or critically compromise the housekeeping functions of the cell, depending on the expression efficiency and the nature of the biochemical interactions, and must be assessed case-by-case when developing production strains for biotechnological use. For the current objective, however, we specifically needed a system such as sYFP2 that would not mask the locus-specific features under cellular stress responses, and allow us to distinguish relatively subtle differences between the generated strains. In accordance, the collected sYFP2 expression profiles (Fig. 2) showed that expression from the four chromosomal sites and the four megaplasmids was very similar throughout the induction phase, and implied that the use of megaplasmids would not provide advantage in relative efficiency, at least under autotrophic conditions (constant light 50 μmol photons m^−2^ s^−1^ and 1% CO_2_). In terms of the chromosomal integration, these observations were in line with earlier reports showing relatively uniform fluorescence between parallel target sites in the *Synechocystis* chromosome [58, 65]. Our data also confirmed that the pCA2.4/I.R.* and pDF-lac2* systems, used for reference, produce clearly higher levels of expression (approx. two- and threefold, respectively) than chromosomal constructs (Figs. 2, 3). The relative fluorescence values, however, deviate significantly from those reported in literature, where the use of pCA2.4 enabled up to 100-fold higher expression in reference to the chromosmal locus *psbA2* (slr1311*), as measured over a 14-days cultivation period under autotrophic conditions (constant light 80–100 μmol photons m^−2^ s^−1^ and 2% CO_2_) [36]. In a similar manner, the RSF1010-derived replicative plasmid pPMQAK1 has been measured to give lower expression than the endogenous small plasmid pCC5.2 even when the integrative construct was not segregated [58]. Although all the existing information consistently supports the use of the plasmid-based systems for maximizing expression efficiencies in *Synechocystis*, the inconsistensies underline the challenges in reliable direct comparison of quantitative data obtained with different expression systems and analytical setups.

Despite the shortcomings of the sYFP2 system to provide stability data under high negative selection, the six-week cultivation carried out without antibiotics (Fig. 6) reinforced our view on the replicative pDF-lac2* vector as a relatively robust laboratory tool. Generally, plasmid stability is determined by the frequency at which plasmid-free progeny will arise during cell division, and the strength of the subsequent positive selection in favor of clones that are not subjected to plasmid-induced stress. *Synechocystis* appears not have active partitioning systems to ensure plasmid distribution into the daughter cells, so plasmid maintenance is entirely based on random distribution during cell division, and therefore largely determined by plasmid copy number. It has been estimated that about 15 plasmid copies is sufficient to maintain stability via random partitioning [66], which is in the typical copy number range (~ 10–30 per cell) reported for RSF1010-derived plasmids in *Synechocystis* (Table 2)*.* This is also in agreement with our findings, which show that the measured 2.4 copies of pDF-lac2* per chromosome (Table 2), enable the stable maintenance of the plasmid in the absence of selection pressure over a batch cultivation period of 42 days (Fig. 6). Similar stability has been reported earlier for the RSF1010-based vector pSEVA251 in *Synechocystis* cells grown in batch cultures for up to 16 days [41], further implying that these plasmids are not spontaneously lost from the cell population simply by the exclusion of antibiotics. With the realization that the use of plasmids is generally restricted by the relative instability in comparison to genomic constructs, particularly when the use of antibiotics is not feasible as in large-scale biotechnological production systems, further studies are needed to evaluate alternative expression constructs under different selective conditions. *Synechocystis* naturally supports the maintenance of the endogenous megaplasmids with a repertoir of specific toxin/antitoxin (TA) systems, that induce post-segregational killing of plasmid-free cells [57, 67]. Such strategy could perhaps be used to promote the positive selection of RSF1010-derived replicative plasmids in the absence of antibiotics in *Synechocystis* [68], and characterized in combination with potentially toxic expression systems that counteract the effect by inducing plasmid instability. The preparative use of these plasmids can be further improved by the inactivation of the conjugation-associated *mobA* system that enhances extraction yields of intact plasmid dsDNA and thus the in vitro cloning efficiency [69, 70], whenever natural transformation is used for generating the strains.

In parallel to the fluorescence expression profiles (Fig. 2) of the nine genomic integration strains (Table 4) and the replicative construct pDF-lac2*, we characterized the corresponding relative copy numbers of the replicons by RT-qPCR (Fig. 5, Additional file 1: Table S5). This enabled the comparison between the expression levels and *syfp2* gene dosage between the targeted loci in the chromosome (slr1311*, *slr0944*, slr0168*, *sll0058*), the megaplasmids (pSYSM/*slr5037-slr5038*, pSYSX/*slr6037*, pSYSA/*slr7023*, pSYSG/*slr8030*), the small plasmid pCA2.4/I.R.* and pDF-lac2*. To our knowledge, such direct and comprehensive comparison has not been conducted and presented in any previous study in *Synechocystis*. Instead, the conclusions now perpetuated in literature relate acquired expression data with previously published copy number information [36, 58] (see Tables 1 and 2). As seen from our two datasets, the copy numbers (Fig. 5) are fairly consistent with the sYFP2 expression profiles between the chromosomal loci (Figs. 2A–D, 3) and the four megaplasmids (Figs. 2E–H, 3). As for the the native plasmid pCA2.4* (Fig. 2I) and the pDF-lac2*-based system (Fig. 2J), both have clearly higher expression levels and replicon copy numbers in comparison to the chromosomal sites and the megaplasmids (Fig. 3). However, there is no perfect correlation between the datasets, as observed for pDF-lac2* which has a significantly lower relative copy number than anticipated based on sYFP2 expression, and pSYSA/*slr7023* that shows lower relative fluorescence in reference to the other target loci in the megaplasmids and in the chromosome. Despite this variance, we expect the data to be a more accurate description of the relationships than mere comparison with information in literature (Table 2), and conclude that there is an overall trend througout the data linking increased *syfp2* gene dosage with higher fluorescence levels from the different integrative loci. In general, it is obvious that gene copy number is reflected in the corresponding expression levels (provided that expression does not compromise the well-being of the host) as previously seen for example in increased ethylene production in *Synechocystis* when additional *efe* genes are introduced into the cell [71]. In accordance, the current study now provides a reference to the phenomenon from the perspective of the alternative genomic sites and the RSF1010-derived expression system in *Synechocystis*. Based on the data, however, we cannot rule out the possibility of overlapping impact of genome position effects caused by the surrounding genetic context around the selected integration sites. Besides experimental factors, this could in part explain the fluctuations observed between the expression profiles (Fig. 2) and the copy numbers (Fig. 5), although further investigation is be needed to confirm potential gene-level interactions.

## Summary

This work provides new quantitative data that expands our current understanding on the use of different integrative genomic sites to express heterologous genes in the model cyanobacterium *Synechocystis* sp. PCC 6803. With the most comprehensive set of targets to date, the main findings demonstrate differences in the relative expression efficiencies between integration sites in the chromosome and in the native plasmids in *Synechocystis*, and for the first time, relates this information directly to the corresponding replicon copy numbers. This knowledge can guide the rational selection of most convenient integration strategies in regards to expression levels and relative cloning efficiencies, when weighing between options to engineer this cyanobacterial host for different practical uses.

## Materials and methods

### Reagents and enzymes

Standard molecular biology techniques and commercial kits (Qiagen, Germany) and enzymes (New England BioLabs, USA or Thermo Fisher Scientific, USA) were used for DNA manipulation. Oligonucleotides were purchased from Eurofins MWG Operon (Germany), and larger gene fragments from GeneScript (USA).

### Organisms and standard growth conditions

A glucose tolerant substrain of *Synechocystis* sp. PCC 6803 [72] obtained originally from Professor Aaron Kaplan (Hebrew University of Jerusalem, IL), was used for all the cyanobacterial experiments. The cyanobacterial strains were grown in BG-11 medium [73] buffered with TES-KOH to pH 8.0 at 30 °C under constant light of 50 μmol photons m^−2^ s^−1^ and 1% CO_2_ atmosphere in a growth chamber MLR-351 (Sanyo, Japan) or Algaetron 230 (Photon Systems Instruments, Czech Republic). *Escherichia coli* strain DH5α was used as the host for plasmid propagation. *E. coli* cells were grown in Lysogeny Broth (LB) medium at 37 °C, at 150–200 rpm or on solid agar plates supplemented with 50 µg ml^−1^ spectinomycin (Sp) and 34 µg ml^−1^ chloramphenicol (Cm) when needed.

### Selection of putative target sites based on transcriptomic data

Besides integration targets selected based on annotated functions (see "Results" section), whole-genome microarray data was used for identifying genes with low expression-level changes upon the transition from constant light to fluctuating light and from ambient carbon concentration to 3% CO_2_ in various eletron transfer mutants (Δ*flv1*, Δ*flv2*, Δ*flv1/3*, Δ*flv4,* Δ*fnrL*, Δ*fed7*, Δ*pgr5*, Δ*ndhB*, Δ*ndbA*).

The datasets had been acquired earlier as described in [48, 49] but not previously published in regards to the expression fold changes presented here (Additional file 1: Tables S1 and S2). In the first round of screening, putative target genes annotated as unknown or hypothetical were chosen based on low overall fold change values (log_2_FC < 1.37) and low differential expression in the datasets. The qualifying genes were then compared with genome-wide expression to exclude targets which showed significant response to different environmental conditions (http://cyanoexpress.sysbiolab.eu), contained predicted internal sense or antisense RNAs [74], or were homologous to other cyanobacterial protein with known function (http://genome.annotation.jp/cyanobase/Synechocystis). Based on the microarray, five genes (*sll0710*, *slr0725*, *slr0868*, *ssr0663*, *sll0403*) were ultimately included in the target list (Table 4) for testing.

### Assembly of the integration constructs for sYFP2 expression in *Synechocystis*

In order to introduce the sYFP2 reporter expression cassette (Additional file 1: Fig. S2) into different loci in the *Synechocystis* genome, an integrative expression plasmid pSI1B (Additional file 1: Figs. S1, S2) was developed based on the commercial *E. coli* vector pUC57 (GenScript). The plasmid contains separate multiple cloning sites for the incorporation of the alternative upstream and downstream homologous sequences corresponding to the different sites of integration, which were PCR-amplified from the host genome using the primers listed in Additional file 1: Table S3. The expression module between these sites consists of the promoter P_A1lacO-1_ [75] and the LacI repressor, in addition to SpeI-SalI sites that allow complementary fragments to be introduced using modular assembly system established earlier [76, 47]. The reporter expression cassette subcloned into the plasmid contained the ORF encoding the fluorescent protein sYFP2 [46], placed immediately downstream the RBS from *Synechocystis cpcB* (*sll1577*) [47], followed by a chloramphenicol resistance cassette (CmR) and transcriptional terminators of *rrnB* T1 and *rrnB* T2 from pDF [40]. The assembly of pSI1B is presented in the Additional file 1: Figs. S1 and S2, and the list of all the final integration constructs in the Additional file 1: Table S4.

### Generation of the *Synechocystis* strains

The assembled integration constructs (Additional file 1: Table S4) were amplified in *E. coli*, sequenced, and transformed into WT *Synechocystis* [77] by resuspending 10 ml of freshly grown cell culture (OD_750_ ~ 1) in 0.5 ml BG11 with 1–5 µg plasmid DNA. After o/n incubation in gentle shaking in dark, the cells were plated on BG11 agar, incubated under low indirect light for 24 h, followed by supplementation of Cm beneath the agar at the final concentration 5 µg ml^−1^. After the appearance of individual antibiotic-resistant colonies (typically few weeks at 30 °C constant light 50 μmol photons m^−2^ s^−1^ under 1% CO_2_), the clones were transferred onto secondary plates (10 µg ml^−1^ Cm), and tertiary plates (20 µg ml^−1^ Cm) when necessary. The cells were typically grown for 1–2 weeks followed by colony PCR analysis and preparation of—80 °C freezer stocks. To promote segregation, incubation times were sometimes extended to over four weeks. Generation of the pDF-lac2* reference strain has been described earlier [47].

### Colony PCR verification of the generated *Synechocystis* strains

The generated *Synechocystis* strains were analyzed by colony PCR to confirm segregation (Additional file 1: Figs. S3, S6) using site-specific primer pairs listed in Additional file 1: Table S3. To prepare the PCR templates, samples of antibiotic-resistant cells were first resuspended in 10 µl MQ water, followed by three successive freeze–thaw cycles (5 min at − 80 °C /5 min at + 60 °C, spin-down in between) to induce cell lysis. After centrifugation, 1 µl of the supernatant was used for each PCR reaction (25 µl reactions using DreamTaq™ polymerase and 2 mM of each primer) with the following parameters: 3 min at 98 °C initial denaturation, denaturation at 95 °C for 10 s, annealing at lower primer Tm plus 3 °C, extension at 72 °C for 45 s (25 cycles).

### Quantitative comparison of sYFP2 expression levels in the generated *Synechocystis* strains

To compare the sYFP2 expression levels, the generated *Synechocystis* strains (Table 4) were subjected to rounds of fluorescence analysis using Tecan microplate reader (Tecan infinite 200 PRO) with 495 nm (ex) /535 nm (em) as described earlier [47]. The cells were cultured in Erlenmeyer flasks and induced by the addition of isopropyl-β-D-thiogalactopyranoside (IPTG) to the final concentration of 1 mM at OD_750_ 0.5, followed by transfer onto 96-well plates and analysis (fluorescence and OD_750_) at the specified timepoints. The fluorescence values were always measured in three technical replicates from different number of independent cultures (n = 6, n = 3 or n = 1; see the"Results" section), and used for calculating the average expression levels and standard deviations relative to OD_750_. Typically, all suspension cultures were carried out in the presence of supplemented antibiotics (5 µg ml^−1^ Cm for the integration mutants, and additional 25 µg ml^−1^ Sp for the control strain harboring pDF-lac2*), except for the stability experiment (Fig. 6), which was conducted in the absence of antibiotics. The relative expression rates (Fig. 2; grey dotted lines) representing the initial fluorescent signal increase over the first hours after induction, were calculated based on the linear fit of the three most consistent timepoints between 0 and 6 h after induction, and averaged between the three parallel measurements.

### Stability of expression strains in long-term step-wise batch cultivations

To test the stability of the generated *Synechocystis* strains, the cells were grown in a batch culture series for six weeks in the absence of antibiotics (Fig. 6). The cultures were carried out in 100 ml Erlenmeyer flasks (50 ml volume), and at each round the cells were first diluted to OD_750_ 0.1 with fresh BG-11. After two days of cultivation the optical density was adjusted to 0.5, followed by the induction with IPTG (1 mM), and measurement of sYFP2 fluorescence after six hours of incubation. The fluorescence was measured again after each one-week cultivation period (at this stage the OD_750_ was around 6–7), adjusted to 0.1 and repeated as before. At each round, the axenity of the cultures was confirmed on LB agar plates. At the end of the six-week culture trial the strains were tested again for segregation using colony PCR (Additional file 1: Fig. S6).

### Sample preparation for s*yfp*2 RT- qPCR

For the relative copy number analysis, cell cultures were grown in 25 ml BG11 (50 ml Erlenmeyer flasks) supplemented with appropriate antibiotics (as described above), and incubated at 30 °C with agitation (150 rpm) under a 12 h light (25 μmol photons m^−2^ s^−1^) /12 h dark regimen. The cultures were first inoculated at OD_730_ ~ 0.3 and grown to OD_730_ ~ 2, and then diluted to 0.3 and grown until OD_730_ ~ 1.5. The axenity of the cultures was confirmed on LB agar plates. For each strain, samples (12.5 ml) were collected in duplicates by centrifugation at 4470 g for 8 min at RT. The cell pellets were resuspended in 2 ml storage buffer (NaCl 150 mM, EDTA 1 mM and Tris–HCl 10 mM, pH 8.0) transferred into 2 ml screw-cap tubes and centrifuged at 13,400 g for 5 min (RT). The supernatant was discarded, and cell pellets were stored at − 20 °C until further use. To obtain the DNA template for the RT-qPCR, cell pellets were thawed on ice and resuspended in 150 µl of resuspension buffer (EDTA 10 mM and Tris–HCl 50 mM, pH 8.0) with 0.1 g of 0.2 mm-diameter glass beads (acid washed, Sigma). The cells were disrupted in two consecutive cycles of vortexing (1 min) and incubation on ice (1 min), followed by centrifugation at 13,400 g for 5 min at 4 °C. The supernatants were kept and the quality and integrity was inspected in 1% (w/v) agarose gel performed by standard protocols using TAE buffer. The nucleic acid concentration and purity (the ratios A_260_/A_280_ and A_260_/A_230_) were measured using NanoDrop ND-1000 spectrophotometer (NanoDrop Technologies, Inc.; USA). The concentration of double stranded DNA (dsDNA) was determined for each sample using the Quantifluor® Dye System and measured in the Quantus™ fluorometer, according to the manufacturer’s instructions (Promega, USA). For the RT-qPCR, the dsDNA concentration of all samples was adjusted to 50 ng µl^−1^, and subsequently diluted to 5 ng µl^−1^.

### RT-qPCR for the determination of s*yfp2* relative copy number

The RT-qPCR was performed on Hard-Shell® 384-Well PCR Plates (thin wall, skirted, clear/white) covered with Microseal® B PCR plate sealing film (Bio-Rad, USA). The reactions (10 µl) were manually assembled and contained 0.125 mM of each primer (see Additional file 1: Table S3), 5 µl of iTaq Universal SYBR Green Supermix (Bio-Rad) and 1 µl of template dsDNA (final concentration 5 ng µl^−1^). The PCR protocol used was: 3 min at 95 °C followed by 40 cycles of 30 s at 95 °C, and 30 s at 60 °C. In the end, a melting curve analysis of the amplicons (10 s cycles between 55 and 95 °C with a 0.5 °C increment per cycle) was carried out. Five standard tenfold dilutions of the dsDNA were used to check the relative efficiency and quality of primers. Negative controls (without template dsDNA) were included. The RT-qPCR reactions were performed with three independent experimental trials and technical triplicates of each dsDNA sample in the CFX384 Touch Real-Time PCR Detection System (Bio-Rad). The data obtained were analyzed using the Bio-Rad CFX Maestro™ 1.1 software (Bio-Rad), implementing an efficiency-corrected delta-delta Cq method (ΔΔCq). The *rrn16Sa.b* and *petB* were validated as reference genes for data normalization using the reference gene selection tool available in the Maestro™ software. The *slr0944* (*arsB*) locus was selected as the control condition representing relative expression equal to one. Statistical analysis was performed by means of a one-way ANOVA using the same software, and tests were considered significant if *P* < 0.05.

The size of the PCR products for each gene was verified by agarose gel electrophoresis and, the amplicons were also cloned into the pGEM-T® Easy (Promega) vector according to the manufacturer’s instructions, and the DNA sequence was confirmed by Sanger sequencing (StabVida, Portugal). These experiments were compliant with the MIQE guidelines [78] to promote the effort for experimental consistency and transparency, and to increase the reliability and integrity of the results obtained.

## Supplementary Information

**Additional file 1: Fig. S1**. Integration vector backbone sequence and plasmid map. **Fig. S2.** sYFP2 reporter construct sequence and plasmid map. **Fig. S3.** Colony PCR verification of the generated *Synechocystis* strains. **Fig. S4.** Negative fluorescent control (*Synechocystis* WT) for Fig. 2. **Fig. S5.** Agarose gel of the cell lysates used as template for RT-qPCR. **Fig. S6.** Colony PCR verification of genetic stability after six-week cultivation. **Table S1.** Conditional expression FCs (mRNA) of the selected target gene candidates. **Table S2.** Conditional expression FCs (mRNA) of additional gene targets. **Table S3.** List and descriptions of the PCR primers used in the study. **Table S4. **Integration constructs generated for sYFP2 expression. **Table S5.** Data of the RT-qPCR *syfp2* analysis. **Table S6.** Amplicon-specific parameters in RT-qPCR.

## Data Availability

The raw data and the research material described in the article are available on request.
