# Supplementary material for: Comparison of alternative integration sites in the chromosome and the native plasmids of the cyanobacterium Synechocystis sp. PCC 6803 in respect to expression efficiency and copy number
Source: Microb Cell Fact. 2021 Jul 10;20:130. doi: 10.1186/s12934-021-01622-2 (PMC8272380; doi:10.1186/s12934-021-01622-2)
Supplement: Supplementary file 1 — Additional file 1: Fig. S1. Integration vector backbone sequence and plasmid map. Fig. S2. sYFP2 reporter construct sequence and plasmid map. Fig. S3. Colony PCR verification of the generated Synechocystis strains. Fig. S4. Negative fluorescent control (Synechocystis WT) for Fig. 2. Fig. S5. Agarose gel of the cell lysates used as template for RT-qPCR. Fig. S6. Colony PCR verification of genetic stability after six-week cultivation. Table S1. Conditional expression FCs (mRNA) of the selected target gene candidates. Table S2. Conditional expression FCs (mRNA) of additional gene targets. Table S3. List and descriptions of the PCR primers used in the study. Table S4. Integration constructs generated for sYFP2 expression. Table S5. Data of the RT-qPCR syfp2 analysis. Table S6. Amplicon-specific parameters in RT-qPCR. [file 12934_2021_1622_MOESM1_ESM.pdf]

Additional file 1 for Nagy et al 2021 (MICF)

Comparison of alternative integration sites in the chromosome and the native plasmids of the cyanobacterium *Synechocystis* sp. PCC 6803 in respect to expression efficiency and copy number

Csaba Nagy, Kati Thiel, Edita Mulaku, Henna Mustila, Paula Tamagnini, Eva-Mari Aro, Catarina C. Pacheco, Pauli Kallio

Fig. S1. Integration vector backbone sequence and plasmid map..………………….………..Page 2.
Fig.S2. sYFP2 reporter construct sequence and plasmid map..…………………….........…....Page 3.
Fig. S3. Colony PCR verification of the generated *Synechocystis* strains…………….……....Page 4.
Fig S4. Negative fluorescent control (*Synechocystis* WT) for Fig. 2…………………….……Page 6.
Fig. S5. Agarose gel of the cell lysates used as template for RT-qPCR..……………………..Page 7.
Fig. S6. Colony PCR verification of genetic stability after six-week cultivation…….…….…Page 8.
Table S1. Conditional expression FCs (mRNA) of the selected target gene candidates. Page 9.
Table S2. Conditional expression FCs (mRNA) of additional gene targets……… ...….....Page 10.
Table S3. List and descriptions of the PCR primers used in the study………………………Page 11.
Table S4. Integration constructs generated for sYFP2 expression…………………………..Page 14.
Table S5. Data of the RT-qPCR *syfp2* analysis……………………………………………...Page 15.
Table S6. Amplicon-specific parameters in RT-qPCR………………………………………Page 16.

References……………………………………………………………………………Page 17.

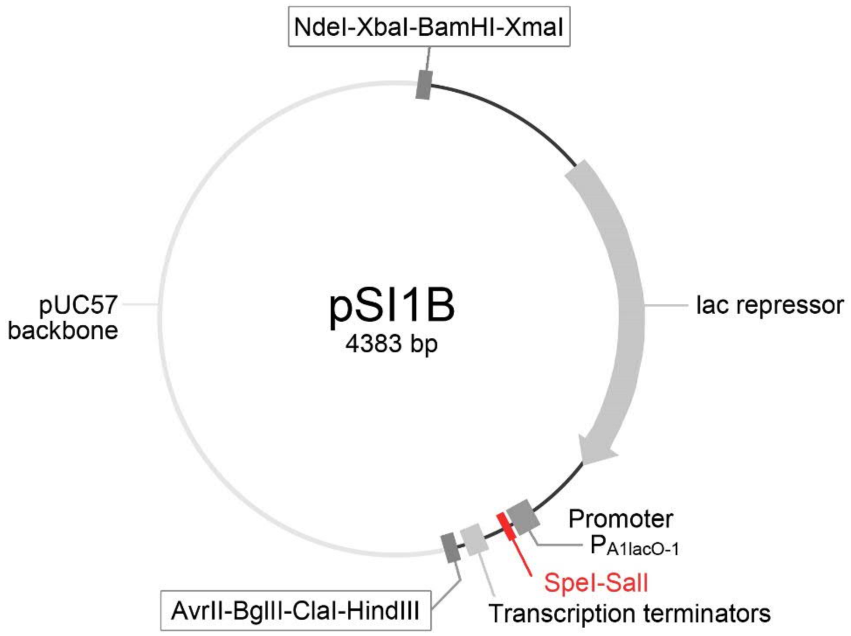

CATATGTTTTCTAGAATTGGATCCAAACCCGGGAACAAGAAGCCATGAAAACCGCCACTGCGCCGTTACCACCGCTGCGT
TCGGTCAAGGTTGTGGACCAGTTGCGTGACGGCAGTTACGCTACTTGCATTACAGCTTACGAACCGAACGAGGCTTATGT
CCACTGGGTTCGTGCCTTTGTACACTCCCGGCATCCGCTTACAGACAAGCTGTGACCGTCTCCGGGAGCTGCATGTGTCA
GAGGTTTTCACCGTCATCACCGAAACGCGCGAGGCAGCAGATCAATTCGCGCGCGAAGGCGAAGCGGCATGCATTTACGT
TGACACCATCGAATGGTGCAAAACCTTTCGCGGTATGGCATGATAGCGCCCGGAAGAGAGTCAATTCAGGGTGGTGAATG
TGAAACCAGTAACGTTATACGATGTCGCAGAGTATGCCGGTGTCTCTTATCAGACCGTTTCCCGCGTGGTGAACCAGGCC
AGCCACGTTTCTGCGAAAACGCGGGAAAAAGTGGAAGCGGCGATGGCGGAGCTGAATTACATTCCCAACCGCGTGGCACA
ACAACTGGCGGGCAAACAGTCGTTGCTGATTGGCGTTGCCACCTCCAGTCTGGCCCTGCACGCGCCGTCGCAAATTGTCG
CGGCGATTAAATCTCGCGCCGATCAACTGGGTGCCAGCGTGGTGGTGTCGATGGTAGAACGAAGCGGCGTCGAAGCCTGT
AAAGCGGCGGTGCACAATCTTCTCGCGCAACGCGTCAGTGGGCTGATCATTAACTATCCGCTGGATGACCAGGATGCCAT
TGCTGTGGAAGCTGCCTGCACTAATGTTCCGGCGTTATTTCTTGATGTCTCTGACCAGACACCCATCAACAGTATTATTT
TCTCCCATGAAGACGGTACGCGACTGGGCGTGGAGCATCTGGTCGCATTGGGTCACCAGCAAATCGCGCTGTTAGCGGGC
CCATTAAGTTCTGTCTCGGCGCGTCTGCGTCTGGCTGGCTGGCATAAATATCTCACTCGCAATCAAATTCAGCCGATAGC
GGAACGGGAAGGCGACTGGAGTGCCATGTCCGGTTTTCAACAAACCATGCAAATGCTGAATGAGGGCATCGTTCCCACTG
CGATGCTGGTTGCCAACGATCAGATGGCGCTGGGCGCAATGCGCGCCATTACCGAGTCCGGGCTGCGCGTTGGTGCGGAT
ATCTCGGTAGTGGGATACGACGATACCGAAGACAGCTCATGTTATATCCCGCCGTTAACCACCATCAAACAGGATTTTCG
CCTGCTGGGGCAAACCAGCGTGGACCGCTTGCTGCAACTCTCTCAGGGCCAGGCGGTGAAGGGCAATCAGCTGTTGCCCG
TCTCACTGGTGAAAAGAAAAACCACCCTGGCGCCCAATACGCAAACCGCCTCTCCCCGCGCGTTGGCCGATTCATTAATG
CAGCTGGCACGACAGGTTTCCCGACTGGAAAGCGGGCAGTGAGCGCAACGCAATTAATACTAGCGAGCGGATACATATTT
GAATGTATTTAGAAAAATAAACAAATAGGGGTTCCGCGCACATTTCCCCGAAAAGTGCCACCTGACGTCTAAGAAACCAT
TATTATCATGACATTAACCTATAAAAATAGGCGTATCACGAGGCCCTTTCGTCTTCACCTCGAGAAATTTATCAAAAAGA
GTGTTGACTTGTGAGCGGATAACAATGATACTTAGATTCAATTGTGAGCGGATAACAATTTCACACAGAATTCATTAAAG
ACTAGTAAGGTACCCGGTGTCGACTTTCGGCCGATACCATGGTTAGATATCTAAGCTAACTTTAataaaacgaaaggctc
agtcgaaagactgggcctttcgttttatctgttgtttgtAGTACTAGATAACTTAGATaaggccatcctgacggatggcc
tttttgcgtttctaCCTAGGATAAGATCTCATATCGATAACAAGCTT

Fig. S1. Representation of the empty integration vector pSI1B backbone. The pSI1B backbone was created by inserting the above DNA fragment (1967 bp) into the commercial plasmid pUC57 at the NdeI-HindIII site. The original subcloned insert (black font) was ordered as a synthetic fragment, followed by the insertion of the PCR-amplified lac repressor cassette and flanking regions (grey font) from the pDF-lac2* template (1) using XmaI and EcoRI (primers listed in **Table S3.**). The target gene to be expressed (*syfp2*; see **Fig. S2.**) can be subcloned into pSI1B as a SpeI-SalI fragment (red font) directly from the pNiv assembly constructs (2) as described earlier (3). The multiple cloning sites (underlined) at each end allow the insertion of the integration site sequences for homologous recombination in *Synechocystis* (upstream: NdeI, XbaI, BamHI, XmaI; downstream: AvrII, BglII, ClaI, HindIII). The transcription terminators *rrnB* T1 and *rrnB* T2 are shown in lowercase font.

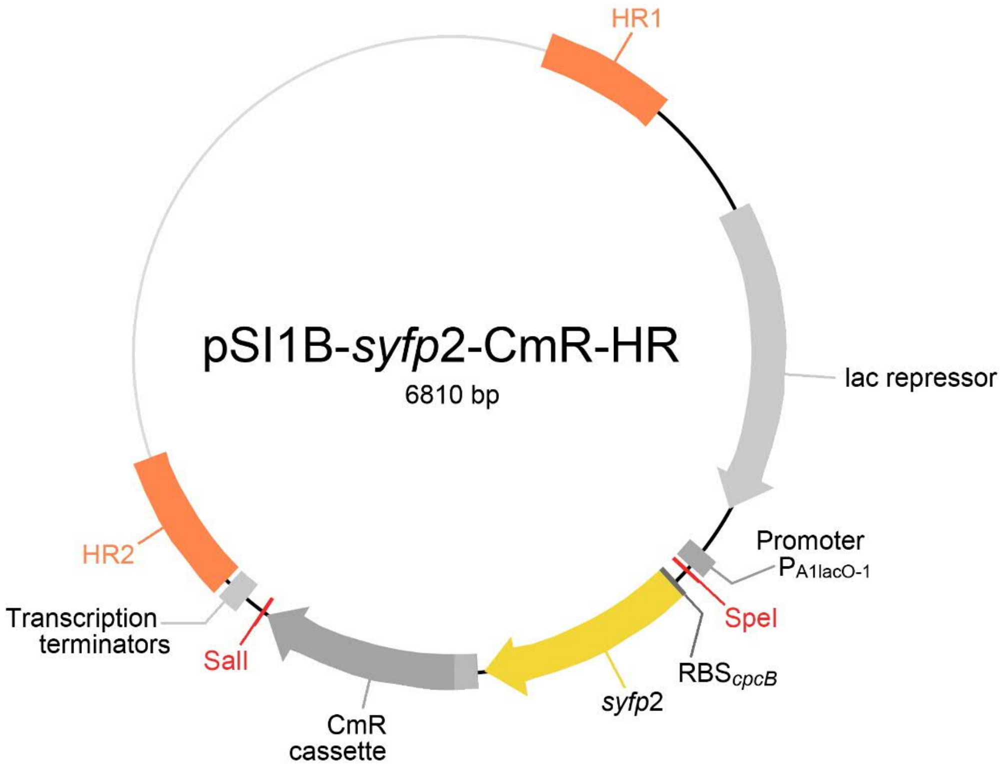

ACTAGTTAATAGAAATAATTTTGTTTAACTTTA**agtcaagtaggagattaattca**ATGCATATGGTGAGCAAGGGCGAGG
AGCTGTTCACCGGGGTGGTGCCCATCCTGGTCGAGCTGGACGGCGACGTAAACGGCCACAAGTTCAGCGTGTCCGGCGAG
GGCGAGGGCGATGCCACCTACGGCAAGCTGACCCTGAAGCTGATCTGCACCACCGGCAAGCTGCCCGTGCCCTGGCCCAC
CCTCGTGACCACCCTGGGCTACGGCGTGCAGTGCTTCGCCCGCTACCCCGACCACATGAAGCAGCACGACTTCTTCAAGT
CCGCCATGCCCGAAGGCTACGTCCAGGAGCGCACCATCTTCTTCAAGGACGACGGCAACTACAAGACCCGCGCCGAGGTG
AAGTTCGAGGGCGACACCCTGGTGAACCGCATCGAGCTGAAGGGCATCGACTTCAAGGAGGACGGCAACATCCTGGGGCA
CAAGCTGGAGTACAACTACAACAGCCACAACGTCTATATCAGTTCCACCGCCGACAAGCAGAAGAACGGCATCAAGGCCA
ACTTCAAGATCCGCCACAACATCGAGGACGGCGGCGTGCAGCTCGCCGACCACTACCAGCAGAACACCCCCATCGGCGAC
GGCCCCGTGCTGCTGCCCGACAACCACTACCTGAGCTACCAGTCCAAGCTGAGCAAAGACCCCAACGAGAAGCGCGATCA
CATGGTCCTGCTGGAGTTCGTGACCGCCGCCGGGATCACTCTCGGCATGGACGAGCTGTACAAGTAAGCTAGCGTTGATC
GGGCACGTAAGAGGTTCCAACTTTCACCATAATGAAATAAGATCACTACCGGGCGTATTTTTTGAGTTATCGAGATTTTC
AGGAGCTAAGGAAGCTAAAATGGAGAAAAAAATCACGGGATATACCACCGTTGATATATCCCAATGGCATCGTAAAGAAC
ATTTTGAGGCATTTCAGTCAGTTGCTCAATGTACCTATAACCAGACCGTTCAGCTGGATATTACGGCCTTTTTAAAGACC
GTAAAGAAAAATAAGCACAAGTTTTATCCGGCCTTTATTCACATTCTTGCCCGCCTGATGAACGCTCACCCGGAGTTTCG
TATGGCCATGAAAGACGGTGAGCTGGTGATCTGGGATAGTGTTCACCCTTGTTACACCGTTTTCCATGAGCAAACTGAAA
CGTTTTCGTCCCTCTGGAGTGAATACCACGACGATTTCCGGCAGTTTCTCCACATATATTCGCAAGATGTGGCGTGTTAC
GGTGAAAACCTGGCCTATTTCCCTAAAGGGTTTATTGAGAATATGTTTTTGTCTCAGCCAATCCCTGGGTGAGTTTCAC
CAGTTTTGATTTAAACGTGGCCAATATGGACAACTTCTTCGCCCCCGTTTTCACGATGGGCAAATATTATACGCAAGGCG
ACAAGGTGCTGATGCCGCTGGCGATCCAGGTTCATCATGCCGTTTGTGATGGCTTCCATGTCGGCCGCATGCTTAATGAA
TTACAACAGTACTGTGATGAGTGGCAGGGCGGGGCGTAATAAGCTAGCGCGGCCGCTCGAGTATGTCGAC

Fig. S2. Representation of the integrative sYFP2 expression constructs used in this work. The insert composed of the sYFP2 coding region (yellow highlight) with an upstream RBS from *Synechocystis cpcB* (*sll1577*) (3) (lowercase bold font), and an independent chloramphenicol resistance cassette (grey highlight) was subcloned into pSI1B (see **Fig. S1.**) as a SpeI-SalI (red font) fragment. The plasmid map shows the relative orientation of the different components in the final construct, flanked by the homologous sequences corresponding to the specific site of integration (orange blocks).

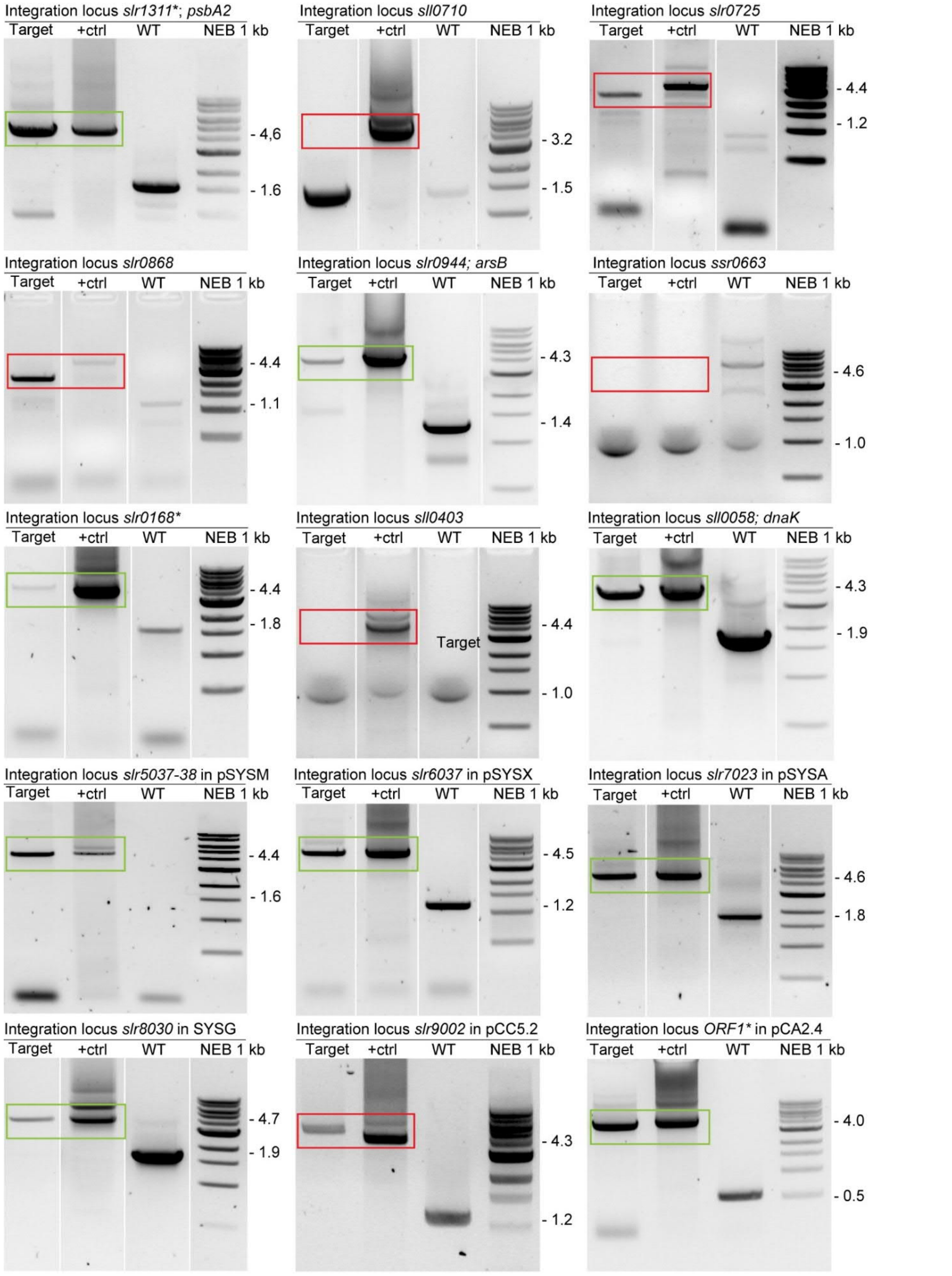

Fig. S3. Colony PCR verification of the generated *Synechocystis* integration site mutants, which harbor the expression cassette for sYFP2 in the designated loci. The integration construct plasmid (**Table S4.**) was used in each case as the template for the positive control (+ctrl), while either genomic DNA extracted from wild-type *Synechocystis* or

crude cell lysate served as the negative control template (WT). The green rectangles indicate the PCR fragments corresponding to the expected size, and the red rectangles depict incorrect bands (i.e. strains excluded from the study), with the calculated sizes for the control fragments on the right. The three integration sites, which have been used earlier and serve here as controls, have been indicated by *. The primers used for the PCR reactions have been specified in the **Table S3**. The strain corresponding to pCB2.4/*ssr9202* is missing from the figure as antibiotic resistant transformants could not be obtained.

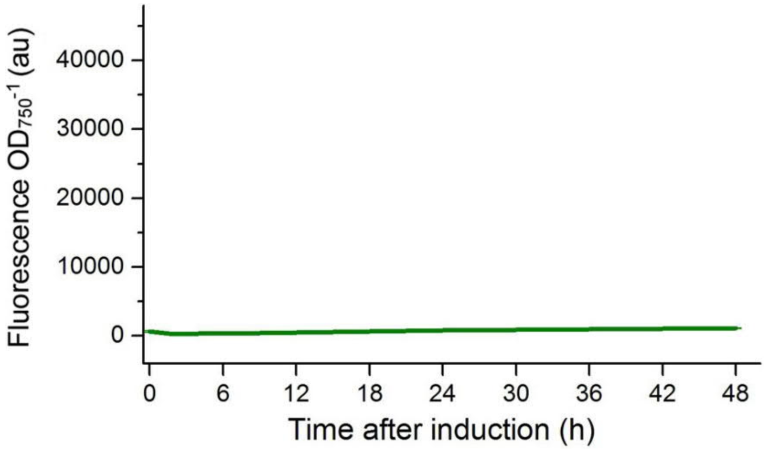

Fig. S4. A representative negative fluorescent control (*Synechocystis* WT) for Fig. 2. An overlay of three parallel fluorescence measurements carried out as described for the mutant strains [495 nm (ex) /535 nm (em)], showing negligible background signal from the WT cells that do not express sYFP2.

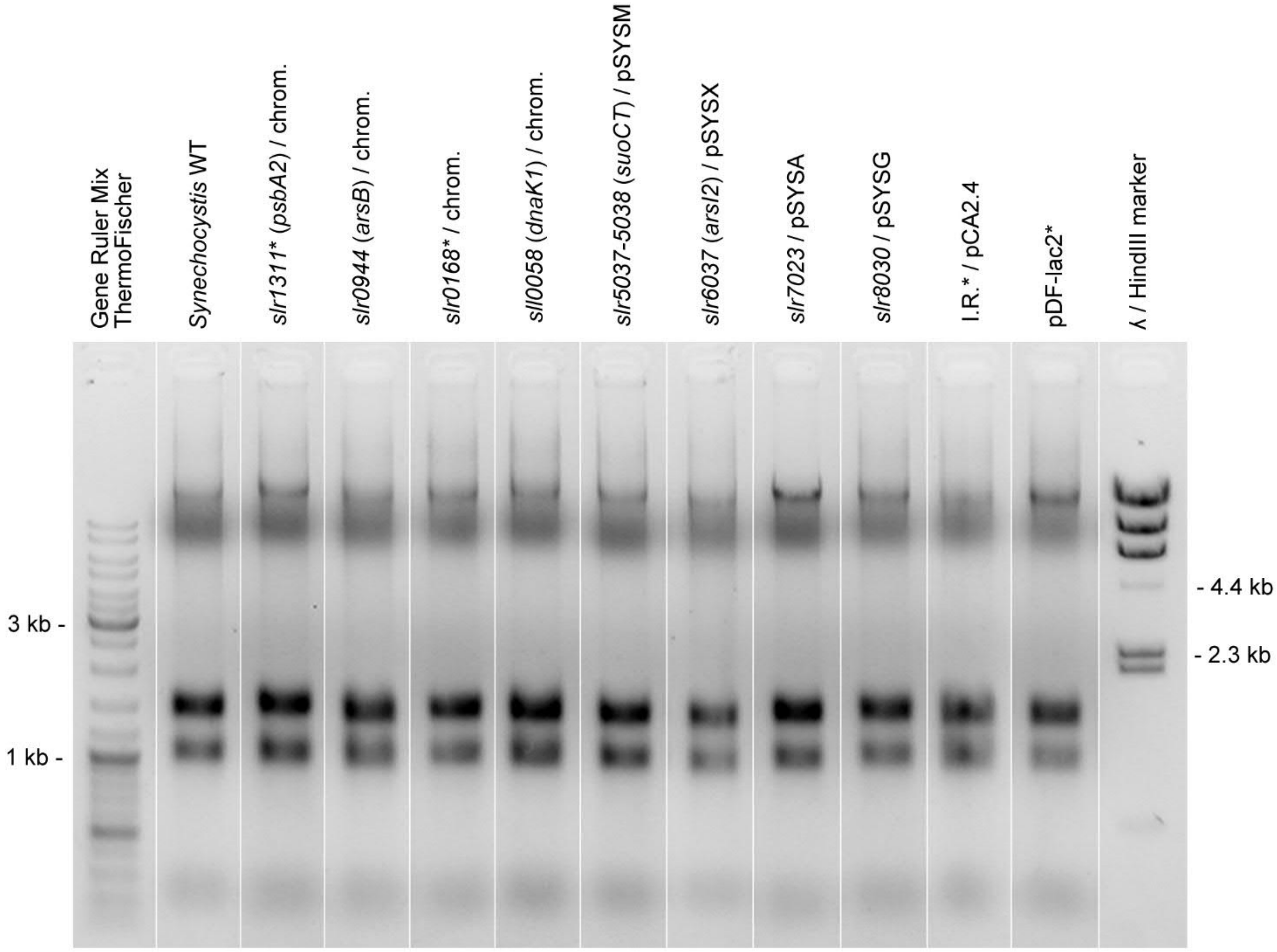

Fig. S5. Confirmation of the quality and integrity of cell lysates obtained from *Synechocystis* WT and strains harboring sYFP2 expression cassette. Agarose gel showing samples corresponding to 100 ng of dsDNA. Molecular Markers – Gene Ruler Mix and ʎ/HindIII (Thermo Fisher Scientific) were used.

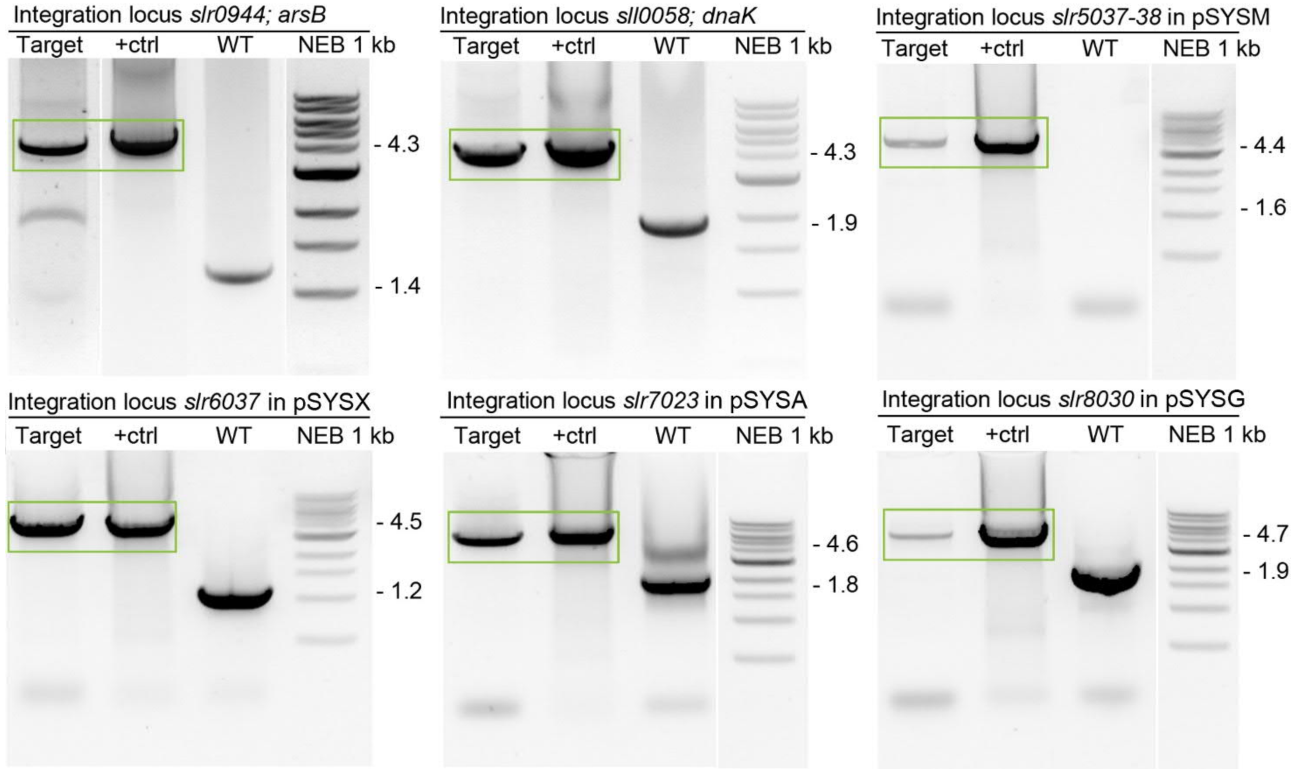

Fig. S6. Colony PCR verification of the generated *Synechocystis* integration site mutants after six-week cultivation without antibiotic selection pressure. The integration construct plasmid (**Table S4.**) was in each case used as the template for the positive control (+ctrl), while wild-type *Synechocystis* crude cell lysate served as the negative control template (WT). The green rectangles indicate the PCR fragments corresponding to the expected size, with the calculated sizes for the control fragments on the right. The primers used for the PCR reactions have been listed in the **Table S3.**.

Table S1. Fold change (log_2FC) and average expression of transcripts of five potential target genes selected based on DNA microarray analysis, performed on *Synechocystis* WT and nine mutant strains carrying deletions in different components of the photosynthetic electron transport chain. The original datasets were acquired as part of earlier studies (4) (5) but the relative expression fold changes listed below have not been presented before. The data reflects the transition from high carbon to low carbon under constant light (HC/LC), the transition from constant light to fluctuating light under ambient CO_2 (LC/FL), and the different expression patterns of the deletion strains in reference to the WT.

	**Comparison**	***ssr0663***	***sll0403***	***slr0725***	***sll0710***	***slr0868***
**Fold change (log_2)**	**WT LC / WT HC**	-0.35	-0.15	-0.01	-0.12	0.66
	**WT FL / WT LC**	0.09	-0.38	-0.28	0.14	-0.39
	**Δ*flv1* LC / WT LC**	0.08	-0.39	0.18	-0.02	-0.07
	**Δ*flv2* LC / WT LC**	-0.20	-0.01	-0.35	-0.06	-0.22
	**Δ*flv1/3* LC / WT LC**	-0.09	-0.10	-0.02	-0.61	0.82
	**Δ*flv4* LC / WT LC**	0.03	0.09	-0.11	0.24	0.08
	**Δ*fnrL* LC / WT LC**	0.28	-0.20	-0.18	0.00	0.23
	**Δ*fed7* LC / WT LC**	0.25	-0.26	-0.26	0.00	-0.12
	**Δ*pgr5* LC / WT LC**	-0.05	0.14	0.04	-0.14	-0.25
	**Δ*flv1* HC / WT HC**	-0.37	0.64	0.55	-0.16	1.37
	**Δ*flv1/3* HC / WT HC**	-0.20	-0.15	-0.16	-0.32	0.48
	**Δ*flv4* HC / WT HC**	-0.44	0.22	0.12	-0.12	0.27
	**Δ*ndhB* HC / WT HC**	0.24	0.37	0.03	0.15	-0.49
	**Δ*ndbA* HC / WT HC**	0.22	-0.19	0.32	-0.38	0.16
	**Δ*flv1* FL / WT FL**	0.01	0.11	0.71	-0.53	-0.14
	**Δ*flv1/3* FL / WT FL**	-0.40	0.54	0.09	-0.44	0.05
**AveExpr (log_2) [a]**	**All samples**	5.73	5.97	6.27	6.45	6.50

[a] The AveExpr column gives the average log_2-expression level for that gene across all the arrays in the experiment. AveExpr scale in this experiment: 5.41-18.49 LC: Low carbon (ambient air) and Constant light (50 µmol photons m^{-2} s^{-1}). FL: Low carbon and Fluctuating light (5 min under 15 µmol photons m^{-2} s^{-1} + 30 sec under 500 µmol photons m^{-2} s^{-1}). HC: High carbon (3% CO_2) and Constant light (50 µmol photons m^{-2} s^{-1}). **Strains** (genes inactivated): **Δ*flv1*** (*sll1521*); **Δ*flv2*** (*sll0219*); **Δ*flv1/3*** (*sll1521, sll0550*); **Δ*flv4*** (*sll0217, sll0218, sll0219*); **Δ*fnrL*** (*slr1643*); **Δ*fed7*** (*sll0662*); **Δ*pgr5*** (*ssr2016*); **Δ*ndhB*** (*sll0223*); **Δ*ndbA*** (*slr0851*).

Table S2. Fold change (log_2FC) and average expression of transcripts of two potential target genes selected based on literature and two reference genes (denoted by *) analyzed for *Synechocystis* WT and nine mutant strains carrying deletions in different components of the photosynthetic electron transport chain. The original datasets were acquired as part of earlier studies (4) (5) but the relative expression fold changes listed below have not been presented before. The data reflects the transition from high carbon to low carbon under constant light (HC/LC), the transition from constant light to fluctuating light under ambient CO_2 (LC/FL), and the different expression patterns of the deletion strains in reference to the WT.

	**Comparison**	***slr1311**** **/ *psbA2***	***slr0944*** **/ *arsB***	***slr0168****	***sll0058*** **/ *dnaK1***
**Fold change (log_2)**	**WT LC / WT HC**	0.07	0.07	0.10	0.50
	**WT FL / WT LC**	0.00	-0.07	-0.17	-0.43
	**Δ*flv1* LC / WT LC**	-0.28	-0.68	0.14	0.11
	**Δ*flv2* LC / WT LC**	-0.08	-0.80	0.15	0.09
	**Δ*flv1/3* LC / WT LC**	-0.54	-0.80	0.10	-0.16
	**Δ*flv4* LC / WT LC**	-0.14	-0.92	-0.34	-0.22
	**Δ*fnrL* LC / WT LC**	-0.05	-0.53	-0.32	-0.07
	**Δ*fed7* LC / WT LC**	-0.31	-0.20	-0.20	-0.19
	**Δ*pgr5* LC / WT LC**	0.24	-0.07	0.12	-0.12
	**Δ*flv1* HC / WT HC**	-1.00	-0.31	0.43	0.16
	**Δ*flv1/3* HC / WT HC**	-0.88	-0.04	0.12	0.40
	**Δ*flv4* HC / WT HC**	-0.09	-0.40	0.10	0.29
	**Δ*ndhB* HC / WT HC**	0.09	-0.88	0.14	0.07
	**Δ*ndbA* HC / WT HC**	-0.07	0.14	0.07	0.26
	**Δ*flv1* FL / WT FL**	0.23	-0.42	-0.26	0.28
	**Δ*flv1/3* FL / WT FL**	-0.04	-0.32	0.08	0.48
**AveExpr (log_2) [a]**	**All samples**	17.98	7.17	10.15	11.29

[a] The AveExpr column gives the average log_2-expression level for that gene across all the arrays in the experiment. AveExpr scale in this experiment: 5.41-18.49 LC: Low carbon (ambient air) and Constant light (50 µmol photons m^{-2} s^{-1}). FL: Low carbon and Fluctuating light (5 min under 15 µmol photons m^{-2} s^{-1} + 30 sec under 500 µmol photons m^{-2} s^{-1}). HC: High carbon (3% CO_2) and Constant light (50 µmol photons m^{-2} s^{-1}). **Strains** (genes inactivated): **Δ*flv1*** (*sll1521*); **Δ*flv2*** (*sll0219*); **Δ*flv1/3*** (*sll1521, sll0550*); **Δ*flv4*** (*sll0217, sll0218, sll0219*); **Δ*fnrL*** (*slr1643*); **Δ*fed7*** (*sll0662*); **Δ*pgr5*** (*ssr2016*); **Δ*ndhB*** (*sll0223*); **Δ*ndbA*** (*slr0851*).

Table S3. PCR primers used in this study. The complementary sequences are shown in capital letters, overhangs in lowercase, and the restriction sites used in the subsequent cloning steps are underlined. The primers used for colony PCR verification of the generated strains are marked with the symbol †, and primers used for the RT-qPCR are marked with ‡. The controls used in the study have been indicated by *.

Template	Primer name	Nucleotide sequence (5' → 3')
pDF-lac2*	LacIq_For_XmaI	ttcccgggAACAAGAAGCCATGAAAACCGCCA
	LacIq_rev_EcoRI	ttaatgaattcTGTGTGAAATTGTTATCCGC
*slr1311**	slr1311UP_F_NdeI†	atacatatgATTGCGGCTTTAGCGTTC
(*psbA2*)	slr1311UP_R_BamHI	ataggatccTAATTCCTTATGTATTTGTCGATGTTC
Chrom.	slr1311DWN_F_AvrII	atacctaggACCGCCGTATTCTTGATCTACC
	slr1311DWN_R_BglII†	taagatctCACAGGAGCTTGCTCCCC
sll0710	sll0710UP_Fw_NdeI†	atacatatgATCAGTTAAATCTAACCACTCAAGTT
(N.A.)	sll0710UP_Rev_BamHI	ataggatccGATTACTTTCCAAAATGAGGCG
Chrom.	sll0710DWN_Fw_AvrII	atacctaggCACTTTATATTTCACGTCAGGAAAGA
	sll0710DWN_Rev_HindIII†	ataaagcttTTTCAATTTGCAAAGAGGCTC
slr0725	slr0725UP_FwNdeI†	atacatatgCCCCATGTTCAGAATCTACCC
(N.A.)	slr0725UP_RevBamHI	ataggatccTTTTTATAATCTTTGGGATTCTTTTG
Chrom.	slr0725DWN_FwClaI	tataatcgatTCTTTAGAAAAATGCTCGAAAATG
	slr0725DWN_RevHindIII†	ataaagcttAGTTTGTGGGTTCTGGGAGA
slr0868	slr0868UP_Fw_NdeI†	atacatatgTTTGAACTGGGTATTGCGG
(N.A.)	slr0868UP_Rev_BamHI	ataggatccTATTTTGAGTTTGGACCTGTCTGT
Chrom.	slr0868DWN_Fw_AvrII	atacctaggAGTTGGCAACTACGCTCAAG
	slr0868DWN_Rev_HindIII†	ataaagcttAAAATCGCTCTAATACAAGGCTATG
slr0944	slr0944UP_F_NdeI†	atacatatgGGATGTGAATGGAACAATGG
(*arsB*)	slr0944UP_R_BamHI	ataggatccTGGGAAAAATCAATTTTCACC
Chrom.	slr0944DWN_F_AvrII	atacctaggCCATTGTGGCTTTGTTATTGAC
	slr0944DWN_R_HindIII†	ataaagcttTGAGGGGGCAGAATAAAACTT
ssr0663	ssr0663UP_F_NdeI†	tatcatatgAAAATGAGGGAATGTACCGTCC
(N.A.)	ssr0663UP_RevBamHI	tatggatccAGCTTACTCAAATGGTTGAATTTTTC
Chrom.	ssr0663DWN_FwAvrI	atacctaggATTCAATGACCCAATGGAGC
	ssr0663DWN_R_BglI†	atagatctCGATGGATATCCGTGACG
*slr0168**	slr0168UP_F_NdeI†	atacatatgATGACTATTCAATACACCCCCCT
(N.A.)	slr0168UP_R_BamHI	ataggatccGATCACCGGGGTGACAATT
Chrom.	slr0168DWN_F_AvrII	atacctaggGGCCCCGTATTGCGTAAT
	slr0168DWN_R_HindIII†	ataaagcttACCGTTAAAAATGGCGCA
sll0403	sll0403UP_F_NdeI†	atacatatgCCTTAGCTTGGGGTTTATATCTTTT

(N.A.)	sll0403UP_RevBamHI	ataggatccCTATTACTATGATTATTGGCTTAGCCG
Chrom.	sll0403DWN_Fw_AvrII	atacctaggTTTCCTCATAAATGTCCGGG
	sll0403DWN_R_HindIII†	ataaagcttAGTTTTTCAACAATATGGTAGTGCC
sll0058	sll0058UP_F_NdeI†	atacatatgCTAATCGATCGCTTCATAGTCACT
(*dnaK1*)	sll0058UP_R_BamHI	ataggatccCAAGCAGAAGAAGGACCAATTG
Chrom.	sll0058DWN_F_AvrII	atacctaggTAGCACTTCAAATACTCCGTTACC
	sll0058DWN_R_HindIII†	ataaagcttAGCGGTTAGTGGGGCAAT
slr5037-38	slr5038UP_F_NdeI†	tatcatatgAGGATTTGATGAAGCTATCGGA
(*suoCT*)	slr5038UP_R_BamHI	tatggatccAGAAATAACCAGGTCATTTCCCC
pSYSM	slr5038DWN_F_AvrII	atacctaggATTCAATGACCCAATGGAGC
	slr5038DWN_R_BglII†	atagatctCGATGGATATCCGTGACG
slr6037	slr6037UP_F_NdeI†	tatcatatgTAATTAACGATTCAGCAGTTTGATAAG
(*arsI2*)	slr6037UP_R_BamHI	tatggatccCAACCTTCCTTCACATACTCAATAAC
pSYSX	slr6037DWN_F_AvrII	atacctaggTCGTCCTAGTGAGGTGGTTTTAG
	slr6037DWN_R_BglII†	atagatctTTTGTCCTGTGGTCAATTAATAGTAAT
slr7023	slr7023UP_F_NdeI†	tatcatatgTTACTCCCAAGGCCGAAG
(N.A.)	slr7023UP_R_BamHI	tatggatccGTTGAGCCAGTACATCCACC
pSYSA	slr7023DWN_F_AvrII	atacctaggATATGTGCGCAGGGGCATA
	slr7023DWN_R_HindIII†	ataaagcttTCCCGACTGCGGTAGTTTT
slr8030	slr8030UP_F_NdeI†	tatcatatgATGGTCTGGTCACGTCGAA
(N.A.)	slr8030UP_R_XmaI	tcccgggGATCTACCAAAGCCGCTTCA
pSYSG	slr8030DWN_F_AvrII	atacctaggAGCGATATTGCTGTTGATAGTTCTACT
	slr8030DWN_R_HindIII†	ataaagcttCACTCCCACGGAATCTAAGC
slr9002	slr9002UP_F_NdeI†	tatcatatgAGGGTACTTAGTCCCTGCACC
(*orfA*)	slr9002UP_R_XmaI	tcccgggAGGGGATTATCTTTGCTGGAA
pCC5.2	slr9002DWN_F_BglII	atagatctGCGAAAAAGTGCGATTTTC
	slr9002DWN_R_HindIII†	ataaagcttaaatcttggactattgccagaatt
I.R.*	pCA24UP_F_NdeI†	tttcatatgTGCTAACCCAGTCACTGTTGG
pCA2.4	pCA24UP_R_XmaI	tcccgggTGTCAAAAACTTAGGCGCAA
	pCA24DWN_F_AvrII	tatcctaggTACTGGGAACAGGGGTAAATGA
	pCA24DWN_R_HindIII†	ataaagcttTTCTATGCCTTATCTGGTAAGGGT
ssr9202	pCB24UP_F_NdeI†	tttcatatgAGTTGACGCTTATGATAAAGCTTATCA
(ORF3)	pCB24UP_R_XmaI	tcccgggTTGCATTATCAGTATTAACCGCAT
pCB2.4	pCB24DWN_F_AvrII	tatcctaggTCTGGTCACAGTTTCGGCA
	pCB24DWN_R_ClaI†	aattatcgatAATATTTGTTTGAAGAAAGGCCC

s*yfp*2	sYFP2_F408‡	ATCGACTTCAAGGAGGAC
	sYFP2_R477‡	CTTGATGCCGTTCTTCTGC
petB	SpetB1F‡ [a]	CCTTCGCCTCTGTCCAATAC
Chrom.	SpetB1F‡ [a]	TAGCATTACACCCACAACCC
rrn16Sa.b	BD16SF1‡ [a]	CACACTGGGACTGAGACAC
Chrom.	BD16SR1‡ [a]	CTGCTGGCACGGAGTTAG

N.A.: Not assigned; I.R.*: Intergenic region between *slr9101* and *pCA24_1* (position 227-465 bp) in pCA2.4.

[a] (6)

Table S4. Integration plasmid constructs generated in this study, that allow the insertion of sYFP2 and CmR expression cassettes into the designated target sites in the *Synechocystis* genome.

Target gene	Replicon	pSI1B-integration construct
*slr1311** *psbA2*	Chrom.	pSI1B-slr1311-IS-sYFP2-CmR
sll0710	Chrom.	pSI1B-sll0710-IS-sYFP2-CmR
slr0725	Chrom.	pSI1B-slr0725-IS-sYFP2-CmR
slr0868	Chrom.	pSI1B-slr0868-IS-sYFP2-CmR
slr0944 *arsB*	Chrom.	pSI1B-slr0944-IS-sYFP2-CmR
ssr0663	Chrom.	pSI1b-ssr0663-IS-sYFP2-Cmr
*slr0168**	Chrom.	pSI1b-slr0168-IS-sYFP2-CmR
sll0403	Chrom.	pSI1b-sll0403-IS-sYFP2-CmR
sll0058 *dnaK1*	Chrom.	pSI1b-sll0058-IS-sYFP2-CmR
slr5037-slr5038 *suoCT*	pSYSM	pSI1B-slr5037-IS-sYFP2-CmR
slr6037 *arsI2*	pSYSX	pSI1B-slr6037-IS-sYFP2-CmR
slr7023	pSYSA	pSI1B-slr7023-IS-sYFP2-CmR
slr8030	pSYSG	pSI1B-slr8030-IS-sYFP2-CmR
slr9002 *orfA*	pCC5.2	pSI1B-slr9002-IS-sYFP2-CmR
I.R.*	pCA2.4	pSI1B-pCA2.4A-IS-sYFP2-CmR
ssr9202 ORF3	pCB2.4	pSI1B-pCA2.4B-IS-sYFP2-CmR

I.R.*: Intergenic region between *slr9101* and *pCA24_1* (position 227-465 bp) in pCA2.4 (7). The target genes used as controls in the study have been indicated by *.

Table S5. Quantification and analysis of samples by absorbance (Nanodrop) and fluorescent dye based system (Quantus fluorometer), and s*yfp*2 relative copy number determined by RT-qPCR (CFX-384). The values represent the average ± standard deviation that have been calculated from three independent replicate cultures (n=3) for each strain. The control strains have been indicated by *.

	Nanodrop			Quantus fluorometer	CFX-384 s*yfp*2 relative copy number	
**Integration sites / strains**	**[nucleic acids] (ng µl^{-1})**	**A_{260}/A_{280}**	**A_{260}/A_{230}**	**[dsDNA] (ng µl^{-1})**	**ΔΔCq [a]**	***P*-value**
*slr1311** (*psbA2*) Chrom.	674.0 ± 21.0	1.54 ± 0.10	0.62 ± 0.05	66.67 ± 18.15	1.10 ± 0.08	0.419
slr0944 (*arsB*) Chrom.	643.7 ± 116.7	1.53 ± 0.08	0.60 ± 0.03	86.67 ± 34.08	1.00 ± 0.07	N.A.
*slr0168** Chrom.	651.7 ± 42.6	1.55 ± 0.05	0.67 ± 0.06	69.67 ± 3.51	1.32 ± 0.10	0.058
sll0058 (*dnaK1*) Chrom.	731.4 ± 87.0	1.53 ± 0.10	0.70 ± 0.03	97.67 ± 50.65	1.17 ± 0.06	0.151
slr5037-5038 (*suoCT*) pSYSM	691.8 ± 91.8	1.54 ± 0.12	0.66 ± 0.05	95.00 ± 47.57	1.72 ± 0.20	0.017
slr6037 (*arsI2*) pSYSX	839.3 ± 264.2	1.51 ± 0.05	0.60 ± 0.07	90.00 ± 11.14	1.85 ± 0.12	0.003
slr7023 pSYSA	616.4 ± 97.1	1.56 ± 0.08	0.62 ± 0.09	68.67 ± 6.11	1.61 ± 0.18	0.023
slr8030 pSYSG	674.4 ± 201.9	1.54 ± 0.06	0.68 ± 0.11	73.00 ± 6.08	1.15 ± 0.03	0.147
I.R.* pCA2.4	631.3 ± 49.4	1.55 ± 0.09	0.66 ± 0.09	80.00 ± 12.49	5.36 ± 0.36	0.000
pDF-lac2*	680.9 ± 118.0	1.55 ± 0.02	0.68 ± 0.02	82.00 ± 11.36	2.39 ± 0.17	0.001
WT	674.0 ± 21.0	1.54 ± 0.10	0.62 ± 0.05	66.67 ± 18.15	N.D.	N.A.

N.D.: not detected; N.A.: not applicable; I.R.*: Intergenic region between *slr9101* and *pCA24_1* (position 227-465 bp) in pCA2.4 (7).

[a] Relative copy numbers of s*yfp*2 were normalized in reference to the endogenous genes *petB* and *rrn16S* in the chromosome. The *slr0944* (*arsB*) locus was chosen as control condition with a relative expression equal to one.

Table S6. Amplicon sizes and parameters derived from RT-qPCR data analysis.

gene	amplicon size (bp)	amplicon Tm (°C)	NTC** (Cq)	amplification efficiency (E)	R^2	slope	y-interception
*syfp*2	69	81	N.D.	90.7	0.999	-3.568	10.255
*petB**	179	82	N.D.	90.5	0.997	-3.573	10.633
*rrn16Sa.b**	190	83.5	>30 [a]	90.4	0.996	-3.575	10.827

**: no template control; *: used as reference genes; N.D.: not detected.

[a] acceptable - amplification in the NTC was detected more than 5 cycles after the standard dilution with lowest concentration.

References

1. Guerrero F, Carbonell V, Cossu M, Correddu D, Jones PR. Ethylene synthesis and regulated expression of recombinant protein in *Synechocystis* sp. PCC 6803. PLoS One. 2012;7(11):e50470.
2. Zelcbuch L, Antonovsky N, Bar-Even A, Levin-Karp A, Barenholz U, Dayagi M, et al. Spanning high-dimensional expression space using ribosome-binding site combinatorics. Nucleic Acids Res. 2013;41(9):e98.
3. Thiel K, Mulaku E, Dandapani H, Nagy C, Aro E-M, Kallio P. Translation efficiency of heterologous proteins is significantly affected by the genetic context of RBS sequences in engineered cyanobacterium *Synechocystis* sp. PCC 6803. Microb Cell Fact. 2018;17(1):34.
4. Mustila H, Allahverdiyeva Y, Isojärvi J, Aro E-M, Eisenhut M. The bacterial-type [4Fe-4S] ferredoxin 7 has a regulatory function under photooxidative stress conditions in the cyanobacterium *Synechocystis* sp. PCC 6803. BBA - Bioenergetics. 2014;1837(8):1293-304.
5. Mustila H, Paananen P, Battchikova N, Santana-Sánchez A, Muth-Pawlak D, Hagemann M, et al. The flavodiiron protein Flv3 functions as a homo-oligomer during stress acclimation and is distinct from the Flv1/Flv3 hetero-oligomer specific to the O_2 photoreduction pathway. Plant Cell Physiol. 2016;57(7):1468-83.
6. Pinto F, Pacheco CC, Ferreira D, Moradas-Ferreira P, Tamagnini P. Selection of suitable reference genes for RT-qPCR analyses in cyanobacteria. PLoS One. 2012;7(4):e34983.
7. Armshaw P, Carey D, Sheahan C, Pembroke JT. Utilising the native plasmid, pCA2.4, from the cyanobacterium *Synechocystis* sp. strain PCC6803 as a cloning site for enhanced product production. Biotechnol Biofuels. 2015;8:201.
